# *In Silico* Exploration of Ayurvedic Extract for Targeting Diabetes Mellitus and Non-alcoholic Fatty Liver Disease *via* GPR120 and GPR40

**DOI:** 10.2174/0115733998380420250411073427

**Published:** 2025-06-12

**Authors:** Priyanka Sharma, Kalicharan Sharma, Anoop Kumar, Mukesh Nandave

**Affiliations:** 1 Department of Pharmacology, Delhi Pharmaceutical Sciences and Research University, New Delhi, 110017, India;; 2 Department of Pharmaceutical Chemistry, ISF College of Pharmacy, Moga, Punjab, 142001, India

**Keywords:** Molecular docking, homology modeling, network pharmacology, diabetes mellitus, NAFLD, ayurvedic herbs

## Abstract

**Introduction:**

Type 2 Diabetes Mellitus (T2DM) is the most prevalent form of hyperglycemia, often coexisting with Non-Alcoholic Fatty Liver Disease (NAFLD), as both share disrupted glucose and lipid metabolic pathways. The study aimed to explore the antidiabetic and hepatoprotective potential of active phytoconstituents from *Tinospora cordifolia*, *Phyllanthus niruri*, and *Picrorhiza kurroa*, focusing on their interaction with GPR120 and GPR 40 receptors through network pharmacology and molecular docking approaches.

**Method:**

A combined extract of *T. cordifolia*, *P. niruri*, and *P. kurroa* was prepared following the Ayurvedic pharmacopoeia standards. Phytoconstituents were identified using High-Performance Thin-Layer Chromatographic (HPTLC) and mass spectroscopy. Network pharmacology analysis predicted mechanisms of action involving PI3-kinase, Protein Kinase C, and PI3K-Akt signaling pathways, targeting GPR120 and GPR40. Molecular docking was conducted for 31 identified compounds, and pharmacokinetic (ADMET) properties of the key hits were evaluated.

**Results and Discussion:**

Molecular docking identified six compounds with strong binding affinities to GPR 120 and 40. Among these, ferulic acid, caffeic acid, and cinnamic acid exhibited significant binding to GPR40 with docking scores of -10.68, -9.991, and -7.580 kcal/mol, respectively. Similarly, veronicoside, malic acid, and corilagin demonstrated strong interaction with GPR120, with docking scores of -8.95, -7.32, and -9.21 kcal/mol. The combined extract contained cinnamic acid and corilagin as major phytoconstituents, supported by favourable ADMET properties. The analysis highlighted their role in modulating glucose and lipid metabolism *via* key signaling pathways, corroborating their antidiabetic and hepatoprotective potential.

**Conclusion:**

This study identifies cinnamic acid and corilagin as promising candidates for natural therapeutics targeting T2DM and NAFLD. The translational potential of *T. cordifolia*, *P. niruri*, and *P. kurroa* provide further experimental validation to confirm their clinical efficacy in modulating metabolic pathways.

## 1 INTRODUCTION

Hyperglycemia is a hallmark of the metabolic disease diabetes mellitus. It is a group of diseases that affect how well your body regulates blood sugar levels. Our health depends on glucose since it is a vital energy source for the cells that make up our muscles and tissues. Recent statistics indicate that there are currently 425 million people with diabetes worldwide, and that number could rise to 629 million or more by the year 2045 [1]. Increased dietary fat intake raises the body's level of fat reserves, which is a known risk factor for metabolic disorders, such as Type 2 Diabetes Mellitus (T2DM), hypertension, coronary artery disease, Non-Alcoholic Fatty Liver Disease (NAFLD), and polycystic ovarian disease [2-7]. There is no denying the connection between NAFLD and T2DM. Recent studies have highlighted NAFLD as a predictor of diabetes development and vice versa, indicating that one condition functions as a potential contributing factor for the other. NAFLD remains challenging to address without causing any side effects, and this difficulty increases when it is combined with diabetes. The rising incidence of type-2 diabetes linked to NAFLD represents one of the major global health challenges today [8-12]. To diagnose early metabolic disorders, particularly insulin resistance, unique problem-solving techniques are urgently required. The preferred treatment for T2DM linked with NAFLD is a synthetic medication, but long-term side effects remain a concern. Due to the greater effectiveness of herbal-based pharmacological therapy in treating diabetes mellitus and its lower risk of adverse effects, health experts have become increasingly interested in it [13-23].

G-protein-coupled receptors (GPCRs) appear to play a key role in liver metabolism and insulin resistance by influencing a variety of signaling pathways in targeting tissues or organs [24-39]. Free fatty Acid Receptors (FFARs) are GPCRs that are activated by Free fatty acids (FFAs). Since FFAs are recognized as signaling molecules, FFARs are a promising target for the treatment of obesity and T2DM. GPR40 (also known as FFAR1) and GPR120 (FFAR4) are two of the FFARs that are activated by long-chain fatty acids (C12-C16) [40, 41]. GPR120 activation alleviates insulin resistance by allowing adipogenesis in adipose tissue, allowing a lipid metabolic balance between adipose and hepatic tissues to be maintained [42]. By promoting GLP-1 and cholecystokinin (CCK) secretion from the gut, GPR120 activation may improve pancreatic cell survival and proliferation and stimulate pancreatic insulin secretion indirectly [43]. GPR40 potentiates insulin secretion in response to medium- and long-chain fatty acids. It is reported that GPR40 deficiency was associated with hepatic inflammation and steatosis in low-fat diet-fed mice [44]. According to the WHO, over 150 plants have been successfully utilized to treat diabetes mellitus, and researchers are looking at hypoglycemic plants. One of the most significant medical systems in the world is that of India. Hence, a unique traditional Indian medicinal herb with the ability to correct hepatic steatosis and hyperglycemia is still urgently required for the prevention of diabetes and its hidden complications [45]. An association between non-alcoholic fatty liver disease and hyperglycemia has already been proposed by several researchers (Fig. **1**) [46, 47].

According to the study, GPR120 and GPR40 are G-protein-coupled receptors whose endogenous ligands are medium- and long-chain free fatty acids, and they are thought to play an important physiological role in insulin release and in preventing the progression of liver damage in NASH by eliciting anti-inflammatory effects [48-51]. Interestingly, while reviewing the literature, we selected *Tinospora cordifolia, Phyllanthus niruri, and Picrorhiza kurroa* plants to identify specific chemical constituents targeting GPR120 and 40. In this investigation, samples from *T. cordifolia, P. niruri, *and *P. kurroa* were analyzed for their physicochemical parameters. Additionally, moisture content, microbial load, heavy metal limit tests, and monographic analysis of the plant materials were conducted. A combined hydro-alcoholic extract of *T. cordifolia*, *P. niruri*, and *P. kurroa* was prepared. Moreover, mass spectroscopy was performed for the qualitative presence of phytoconstituents of the respective plant extracts whereas, we quantified certain phytoconstituents. Based on quantification, we predicted the interaction between 22 chemical constituents of *T. cordifolia*, 11 of *P. niruri*, and 16 of *P. kurroa* as ligands for GPR120 and 40. This study delves into the potential of *T. cordifolia*, *P. niruri*, and *P. kurroa* to manage diabetes and fatty liver disease. By combining network pharmacology and molecular docking, we explored how these herbs target key pathways like PI3K-Akt and receptors, such as GPR 120 and GPR 40. Compounds such as cinnamic acid and corilagin emerged as promising candidates with favorable safety profiles. These findings pave the way for developing natural therapies for diabetes and related liver conditions.

### 1.1 Study Design

This study employed an *in-silico* approach to explore the potential of a combined Ayurvedic extract from *T. cordifolia*, *P. niruri*, and *P. kurroa* for managing T2DM and NAFLD. The extract was prepared following Ayurvedic Pharmacopoeia standards, and its key phytoconstituents were identified using High-Performance Thin-Layer Chromatographic (HPTLC) and mass spectrometry. Network pharmacology was utilized to predict the mechanisms of action with a focus on PI3-kinase, Protein Kinase C, and PI3K-Akt signaling pathways, targeting GPR120 and 40. Molecular docking was performed on 31 compounds, and their pharmacokinetic properties were assessed using ADMET(absorption, distribution, metabolism, excretion, and toxicity) analysis to identify safe and effective candidates.

## 2 MATERIAL AND METHODS

### 2.1 Preparation, Standardization, and Phytochemical Characterization of Extracts

#### 2.1.1 Plant Collection and Authentication

Plant materials, including *Tinospora cordifolia* (roots and stems), *Phyllanthus niruri* (leaves), and *Picrorhiza kurroa* (roots and rhizomes), were procured from the Tuticorin District of Tamil Nadu, India. The samples were authenticated by the Council of Scientific and Industrial Research- National Institute of Science Communication and Information Resources (CSIR-NISCAIR), New Delhi.

#### 2.1.2 Extraction and Standardization

The dried and pulverized plant materials (500 g each) were subjected to maceration using a hydro-alcoholic solvent (ethanol: water, 70:30) at room temperature. The extraction yields were as follows: 5.097% for the hydro-alcoholic extract, 2.164% for the chloroform extract, and 1.850% for the n-hexane extract. All plant extracts underwent physicochemical analysis, including assessments of moisture content, microbial load heavy metal limits, and monographic parameters, following the standards outlined in the Ayurvedic pharmacopoeia [52, 53]. The results were confirmed to be within acceptable limits, ensuring the safety and quality of the extracts.

#### 2.1.3 Phytochemical Characterization using HPTLC

High-performance Layer Chromatography (HPTLC) was employed to quantify 6 key bioactive phytoconstituents: cinnamic acid, corilagin, ferulic acid, caffeic acid, malic acid, and veronicoside [54].

#### 2.1.4 Chemicals and Reagents

Reference standards for the targeted phytoconstituents were procured from Sigma Aldrich Chemical Pvt Ltd, Delhi. HPTLC-grade methanol, water, and other analytical-grade solvents were used throughout the study.

#### 2.1.5 Instrumentation and Analytical Conditions

HPTLC analysis was performed on a Shimadzu Nexera X2 system equipped with a quaternary pump (LC-30AD), autosampler (SIL-30AC), and column oven (CTO-20AC). Data acquisition and processing were conducted using LabSolution software (version 6.08). Chromatographic separation was achieved using a Phenomenex Luna C18 (2) 100A column (4.6 x 250 mm, 5.0 μm). After evaluating multiple solvent systems, a mobile phase consisting of ethyl acetate, toluene, and formic acid in the ratio of 5:4:1 v/v was selected. The detection wavelengths for cinnamic acid, caffeic acid, ferulic acid, and veronicoside were set at 254 nm, 273 nm, 273nm, and 260 nm, respectively. The method successfully resolved the peaks of all compounds with high repeatability and precision. The calibration curve for each analyst exhibited strong linearity, confirming the reliability of the method for simultaneous quantification [55].

### 2.2 Preparation of Standard and Sample Solutions

#### 2.2.1 Standard Solutions

Stock solutions of the reference compounds (cinnamic acid, ferulic acid, caffeic acid, and veronicoside) were prepared at a concentration of 1 mg/mL by accurately dissolving 10 mg of each standard in 10 mL methanol. Calibration curves were generated using five different concentrations, each injected in triplicate.

#### 2.2.2 Sample Solutions

Hydro-alcoholic extracts were prepared by dissolving 50 mg of extract in 10 mL methanol: water (70:30), followed by sonication at 40 kHz frequency and 200 W power for 30 minutes.

The solutions were then filtered through a 0.45 μm syringe filter before injection into the HPTLC system.

### 2.3 Quantitative Analysis

Quantification of phytoconstituents was performed using the external standard calibration method. A strong linear correlation (R^2^ > 0.99) was observed for all reference compounds, confirming the precision of the method. The concentration of each compound in the extracts was calculated based on the calibration curves. Results indicated that ferulic acid and corilagin were the major constituents, with varying amounts of another bioactive compound present.

### 2.4 Mass Spectrometric Analysis

Mass Spectrometric (MS) analysis was conducted at the Sophisticated Analytical Instrumentation Facility (SAIF), Panjab University, Chandigarh. Methanol (MS-grade), water, and formic acid were used as solvents. MS analysis identified additional phytoconstituents beyond those quantified by HPTLC, providing a more comprehensive phytochemical profile.

### 2.5 Homology Modeling

As the crystal structure of GPR120 was unavailable in the Protein Data Bank (PDB), homology modelling was employed to construct its three-dimensional structure. The amino acid sequence of human GPR 120 (UniProt ID: P14672) [20] was retrieved from the Swiss-Prot Database. PSI-Blast analysis identified 4PYP as the most suitable template due to its 65% sequence identity. The structure was modelled using MODELLER 9v9.19, generating multiple models ranked by an internal scoring function. The best model was validated using Ramachandran plot analysis and PROCHECK, which confirmed that >90% of residues were in allowed regions, ensuring their suitability for docking studies [56-58].

### 2.6 Molecular Docking

Molecular docking studies were conducted to investigate the binding interactions between the identified phytoconstituents and the GPR120 and 40 receptors. Ligands were prepared using the LigPrep tool from the Schrodinger suite, optimizing their geometry and stereochemistry. The crystal structure of the GPR40 receptor (PDB ID: 4PHU) was retrieved from the protein data bank (PDB), while the homology-modeled GPR120 structure was used as the docking target. Proteins were pre-processed using Maestro’s Protein Preparation Wizard, where water molecules and heteroatoms were removed to enhance docking accuracy. Docking simulations were conducted using the Glide XP (Extra Precision) module, and the results were ranked based on docking scores, hydrogen bonding, and hydrophobic interaction.

### 2.7 ADMET and MM/GBSA Study

To access the pharmacokinetic properties and toxicity of the top-ranked ligands, Swiss-ADME (http://www.swissadme.ch) was used. This tool enabled the evaluation of key ADMET (absorption, distribution, metabolism, excretion, and toxicity) parameters, including drug-likeness, lipophilicity, Topological Polar Surface Area (TPSA), and bioavailability score. The MM-GBSA (Molecular Mechanics/Generalized Born Surface Area) approach was used to estimate binding affinities and analysis of the protein-ligand complexes. The Maestro 11.6 software module was employed for this calculation, utilizing glide XP docking pose viewer (PV) files and sampling minimization protocol based on a continuum model. The OPLS3e force field was applied to maintain protein flexibility, while VSGB 2.0 was used as a dielectric solvent model, corporating empirical modifications for π-stacking and H-bond interactions. The most stable complex was identified based on its higher negative binding energy.

### 2.8 Network Pharmacology

Network pharmacology was employed to explore the systemic mechanisms of six selected phytoconstituents (cinnamic acid, ferulic acid, caffeic acid, corilagin, malic acid, and veronicoside) in modulating diabetes and NAFLD pathways. The chemical structures of these bioactive compounds were uploaded to the BindingDB database, where potential protein targets were identified based on a similarity score of >0.7 [59, 60]. The corresponding gene names were retrieved from the UniProt Knowledgebase, and the associated signaling pathways were mapped using the KEGG database. Pathway analysis revealed the involvement of these compounds in key regulatory mechanisms, including PI3K-Akt, MAPK, and PKC signaling pathways [61-65]. A comprehensive network of bioactive-target pathways was visualized using Cytoscape 3.7.2, highlighting the pivotal role of these phytoconstituents in modulating glucose and lipid metabolism, improving insulin sensitivity, and reducing hepatic inflammation [66-69].

## 3 RESULTS AND DISCUSSION

### 3.1 Phytochemical Composition and Characterization of Tinospora Cordifolia, Phyllanthus Niruri, and Picrorhiza Kurroa

The hydroalcoholic extract of *T. cordifolia*, *P. niruri*, and *P. kurroa* was prepared, yielding 12%, 7%, and 30%, respectively. Quantitative phytochemical analysis using HPTLC identified six major bioactive constituents: cinnamic acid, veronicoside, corilagin, caffeic acid, malic acid, and ferulic acid, with cinnamic acid and corilagin being the predominant components across the extracts. Mass spectrometry detected a total of 42 phytoconstituents. The extracts complied with pharmacopeial standards for physiochemical properties, with heavy metals and microbial load levels within acceptable limits, as detailed in Supplementary File **1**. The concentration of bioactive compounds in the hydroalcoholic extracts of *T. cordifolia*, *P. niruri*, and *P. kurroa* are presented in Table **1**. Cinnamic acid was quantified in *P. kurroa* at 312.0 µg/100 mg, representing 3.12% of the extract. The regression equation y=0.0037x + 0.0055 demonstrates a strong linear relationship, ensuring the accuracy and reliability of the quantification. Ferulic acid, predominantly found in *T. cordifolia* at 2.75%, had a content of 275.0 µg/100 mg, supported by the regression equation y=0.0041x + 0.0078. Corilagin, the most abundant compound (3.50%) in *Phyllanthus niruri,* was quantified at 350.0 µg/100 mg, with the regression equation y= 0.0052x + 0.0093, indicating good linearity and reproducibility. Caffeic acid, present in *P. niruri* at 1.85%, was quantified at 185.0 µg/100 mg, validated by the regression equation y= 0.0039x + 0.006. Veronicoside, quantified in *P. kurroa* (2.30%), had a content of 230. µg/100 mg, supported by the regression equation y = 0.0045x + 0.0030. Lastly, malic acid quantified at 2.90**%**, *T. cordifolia*, had a content of 290.0 µg/100 mg, with the regression equation y = 0.0042x + 0.0043, ensuring accurate and reliable measurement.

### 3.2 Network Pharmacology Analysis

Network pharmacology offers valuable insights into the intricate mechanisms through which phytoconstituents exert their therapeutic effects, particularly in the context of diabetes and NAFLD. Our findings suggest that the modulation of GPR120 and GPR40 by *T. cordifolia*, *P. niruri*, and *P. kurroa* may contribute to improving insulin sensitivity and lipid metabolism, thereby offering potential therapeutic benefits for both diabetes and NAFLD. The constructed interaction network (Figs. **2** and **3**) highlights the complex relationship between the bioactive compounds and key metabolic pathways, reinforcing the polypharmacological nature of these botanicals. Notably, the PI3K-Akt pathway, which regulates glucose uptake and insulin sensitivity, emerged as a primary target of bioactive compounds from *T. cordifolia*, *P. niruri*, and *P. kurroa* and showed strong associations with the ERK2 signaling pathway, suggesting its role in cell proliferation and differentiation. Additionally, the modulation of MAPK and Protein kinase C (PKC) pathways indicates potential anti-inflammatory and glucose-regulating effects. Our study underscores the relevance of network pharmacology in elucidating the mechanisms underlying the therapeutic effects of medicinal plants. By integrating computational approaches with experimental findings, we provide a comprehensive understanding of how *T. cordifolia*, *P. niruri*, and *P. kurroa* exert their beneficial effects on diabetes and NAFLD. These insights pave the way for further research on the development of target botanical therapeutics for metabolic disorders.

### 3.3 Molecular Docking

Molecular docking studies confirmed the high-affinity binding of phytoconstituents to GPR120 and GPR40, reinforcing their potential as therapeutic agents. Among the six phytoconstituents, ferulic acid, caffeic acid, and cinnamic acid exhibited strong binding affinity to GPR40, with docking scores of 10.68, -9.991, and -7.580 kcal/mol, respectively, as shown in Table **2**. These compounds formed 3-4 hydrogen bonds and 4-6 hydrophobic interactions with key residues, such as Tyr-224, Arg-258, and Ser-187 in the GPR40 binding site. Similarly, veronicoside, malic acid, and corilagin demonstrated favorable interactions with GPR120, forming stable 3-5 hydrogen bonds and 3-5 hydrophobic interactions with key residues, such as Ala-73, Arg-136, and Thr-324. These docking results align with the functional implication observed in network pharmacology. Notably, ferulic acid exhibits enhanced stability within the GPR40 binding pocket, as reflected by the **Δ**G binding value of -44.3965 kcal/mol, suggesting its potential role in glucose metabolism regulation. The detailed 3-D binding interactions of malic acid, cinnamic acid, corilagin, ferulic acid, veronicoside, and caffeic acid, with GPR120 and 40, are illustrated in Fig. (**4**).

#### 3.3.1 On GPR40

Molecular docking investigations were conducted using the Glide module of the Schrodinger 9.6 software (PDB ID; 4PHU). As shown in Fig. (**4a** , **c**), cinnamic acid, caffeic acid, and ferulic acid exhibited strong binding sites of GPR40. These molecules were well accommodated within the receptor cavity and formed stable hydrogen bonds with Tyr 2240; 91 and Arg 2258; 183. To validate the docking approach, the reference ligand docosahexaenoic acid (DHA) was re-docked into the active site of GPR40 and found to occupy the same binding pocket as shown in Figs. (**4b** and **d**) with a root-mean-square deviation (RMSD) of 2.33 A, confirming the reliability of the docking protocol. The superimposition of cinnamic acid, caffeic acid, and ferulic acid with co-crystallized ligand (DHA) revealed a similar binding interaction within the catalytic domain of GPR40, as illustrated in Fig. (**4e**). The docking scores of standard DHA and tested phytoconstituents were comparable, further supporting their potential as GPR40 modulators.

#### 3.3.2 On GPR120

Due to the absence of an experimentally determined crystal structure for GPR120 in the PDB database, homology modeling was performed using MODELLER 9v9.19 software. Docking investigations were then carried out using the Glide module of the Schrodinger 9.6 software. As shown in Figs. (**5a** and **c**), veronicoside, corilagin, and malic acid exhibited strong binding within the active site of GPR120, forming stable hydrogen bonds with key residues, including Ala 73; 75; and Arg 73;136; 70, and Thr 74;324. The reference ligand (DHA) was re-docked into the active site and occupied the same binding pocket (Figs. **5b** and **d**) with a root-mean-square deviation (RMSD) of 0.3 A, confirming the validity of the docking approach. The superimposition of veronicoside, corilagin, and malic acid with co-crystallized ligand (DHA) demonstrated a similar binding interaction within the catalytic domain of GPR120, as depicted in Fig. (**5e**). The docking scores of DHA and the tested phytoconstituents were closely aligned, indicating their potential as GPR120 modulation.

### 3.4 Pharmacokinetic and ADMET Analysis

ADMET predictions further validated the drug-like potential of the selected phytoconstituents. All compounds complied with Lipinski’s Rule of Five, indicating favorable oral bioavailability (>80%), as summarized in Table **3**. Notably, ferulic acid, corilagin, and cinnamic acid demonstrated excellent absorption properties while exhibiting minimal blood-brain barrier permeability. This suggests a reduced risk of off-target central nervous system effects, enhancing their safety profile for diabetes and NAFLD management.

### 3.5 Molecular Mechanics with Generalized Born Surface Area (MM-GBSA) Study

Molecular mechanics with generalized Born surface area (MM/GBSA) studies were conducted to assess the stability of the selected phytoconstituents within the binding pocket of the GPR120 and GPR 40 receptors. Six key phytoconstituents were analyzed using MM/GBSA methods. The results indicated that veronicoside exhibited a binding free energy of -41.0839 kcal/mol, whereas the reference compound, DHA, showed a binding free energy of -38.545 kcal/mol against GPR120. Similarly, caffeic acid demonstrated a binding free energy of -50.344 kcal/mol compared to -41.252 kcal/mol for DHA against GPR40. These findings suggest that the selected phytoconstituents form stable interactions within their respective receptor pockets, as summarized in Table **4**.

## 4 STUDY LIMITATIONS

This study highlights the potential of Ayurvedic extracts in targeting GPR 120 and 40 for diabetes and NAFLD management using *in silico* methods. However, computational models have limitations, as they do not account for metabolism, bioavailability, and dynamic receptor-ligand interactions in a biological system. Additionally, the study focuses on GPR 120 and 40, but other metabolic pathways may also play a role. To confirm these findings, *in vitro* and *in vivo* validation is necessary, as *in silico* prediction alone cannot ensure real-world efficacy [70-75]. Future studies should integrate cell-based assays and animal models to establish pharmacological relevance and clinical applicability.

## CONCLUSION

The integration of phytochemical profiling, network pharmacology, and molecular docking studies highlights the therapeutic potential of *T. cordifolia*, *P. niruri*, and *P. kurroa* in managing diabetes and NAFLD. Six major bioactive compounds, such as cinnamic acid, veronicoside, corilagin, caffeic acid, malic acid, and ferulic acid, exhibited significant interactions with key metabolic pathways, including PI3K-Akt, MAPK, and PKC, which are critical in glucose metabolism, insulin sensitivity, and inflammation regulation. The strong binding affinities of these phytoconstituents to essential receptors, such as GPR120 and 40, further underscored their potential to modulate glucose and lipid metabolism effectively. Additionally, ADMET analysis confirmed favorable drug-like properties, including high oral bioavailability, reinforcing their suitability for therapeutic use. These findings highlight the translational potential of *T. cordifolia*, *P. niruri*, and *P. kurroa* extracts as natural, bioactive-rich candidates for novel interventions targeting diabetes and NAFLD. Their multifaceted mechanism of action positions them as promising agents for both prevention and adjunctive therapy, bridging the gap between traditional herbal medicine and modern pharmacological approaches.

## Figures and Tables

**Fig. (1):**
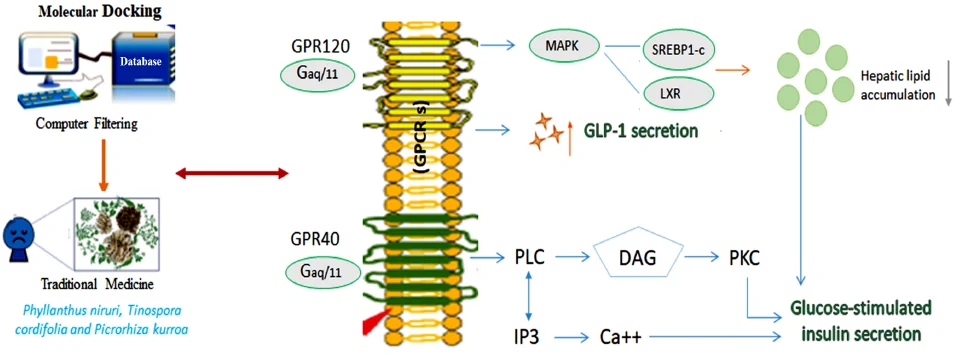
Representation of correlation between T2DM and NAFLD with GPCRs. Abbreviation: LXR: Liver X receptor; MAPK: Mitogen-activated protein kinase; MCFA: Medium-chain fatty acid; SREBP1-c: Sterol regulatory element-binding protein 1; GLP-1: Glucagon-like peptide-1; PLC: phospholipase C; DAG: Diacylglycerol; PKC: Protein kinase C; IP3: Inositol Phosphate 3 metabolic pathways.

**Fig. (2):**
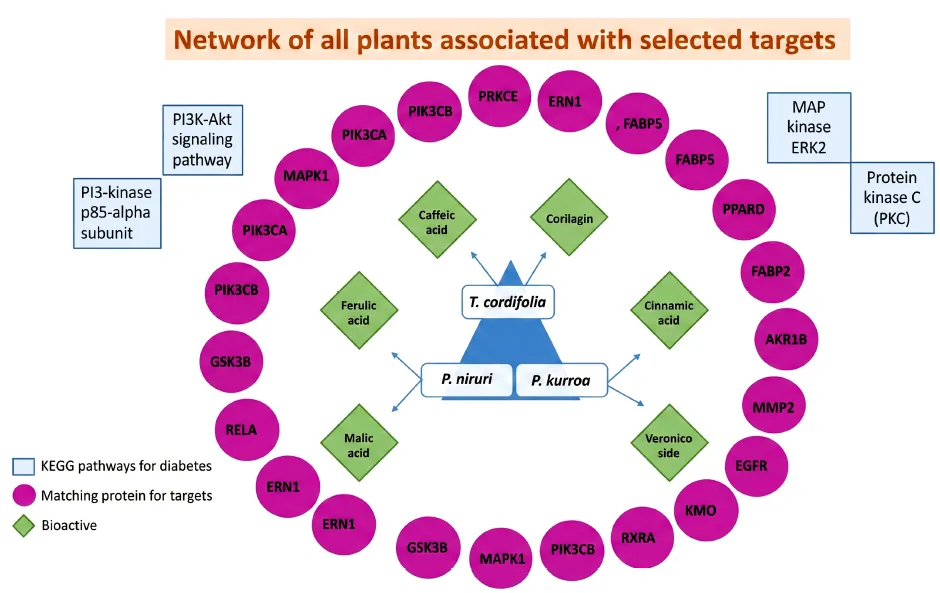
Network of all three botanicals associated with Diabetes and NAFLD pathways. The figure depicts the potential mechanism of all three botanicals to modulate diabetes association with NAFLD pathways through bioactive-target associations.

**Fig. (3):**
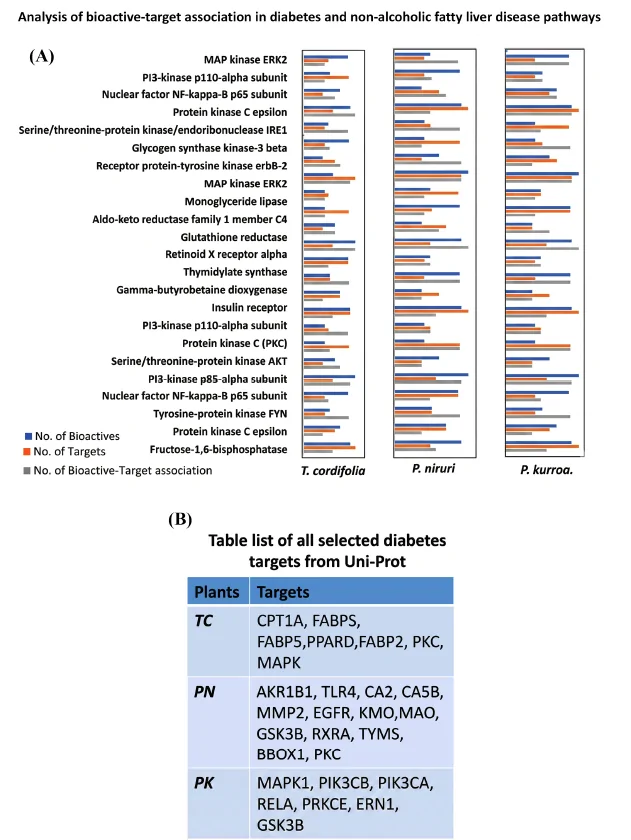
Analysis of bioactive-target associations in diabetes and NAFLD pathways. (**A**) The figure depicts the involvement of TC, PN, and PK in pathways of Diabetes associated with NAFLD. The represented association is several connecting combinations between bioactive and their targets in a particular pathway. (**B**) The table lists all diabetes and NAFLD targets of TC, PN, and PK.

**Fig. (4):**
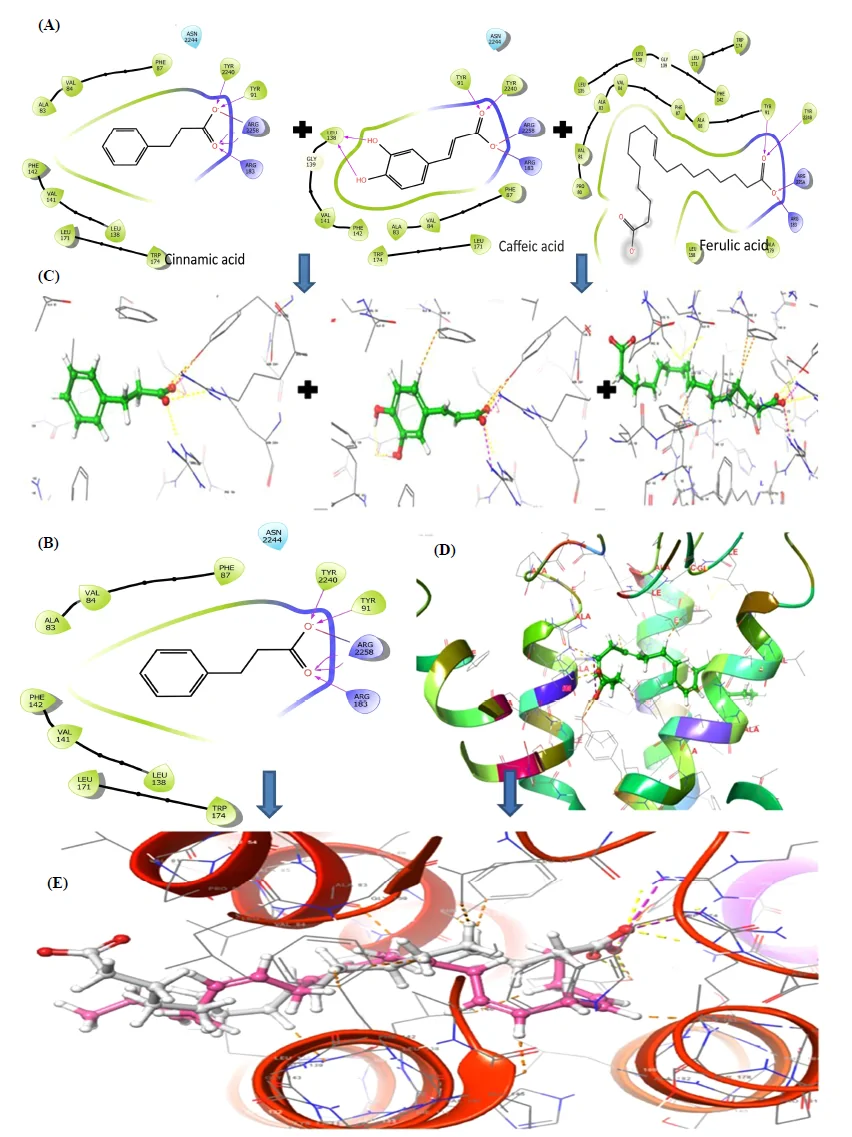
(**A**−**C**) Binding interaction of compound Cinnamic acid, Caffeic acid, and Ferulic acid against GPR40 (PDB ID; 4PHU). (**B**−**D**) Binding interaction of standard drug DHA against GPR40. (**E**) Superimposition of compound cinnamic acid, Caffeic acid, and Ferulic acid (green) with DHA (brown) against GPR40.

**Fig. (5):**
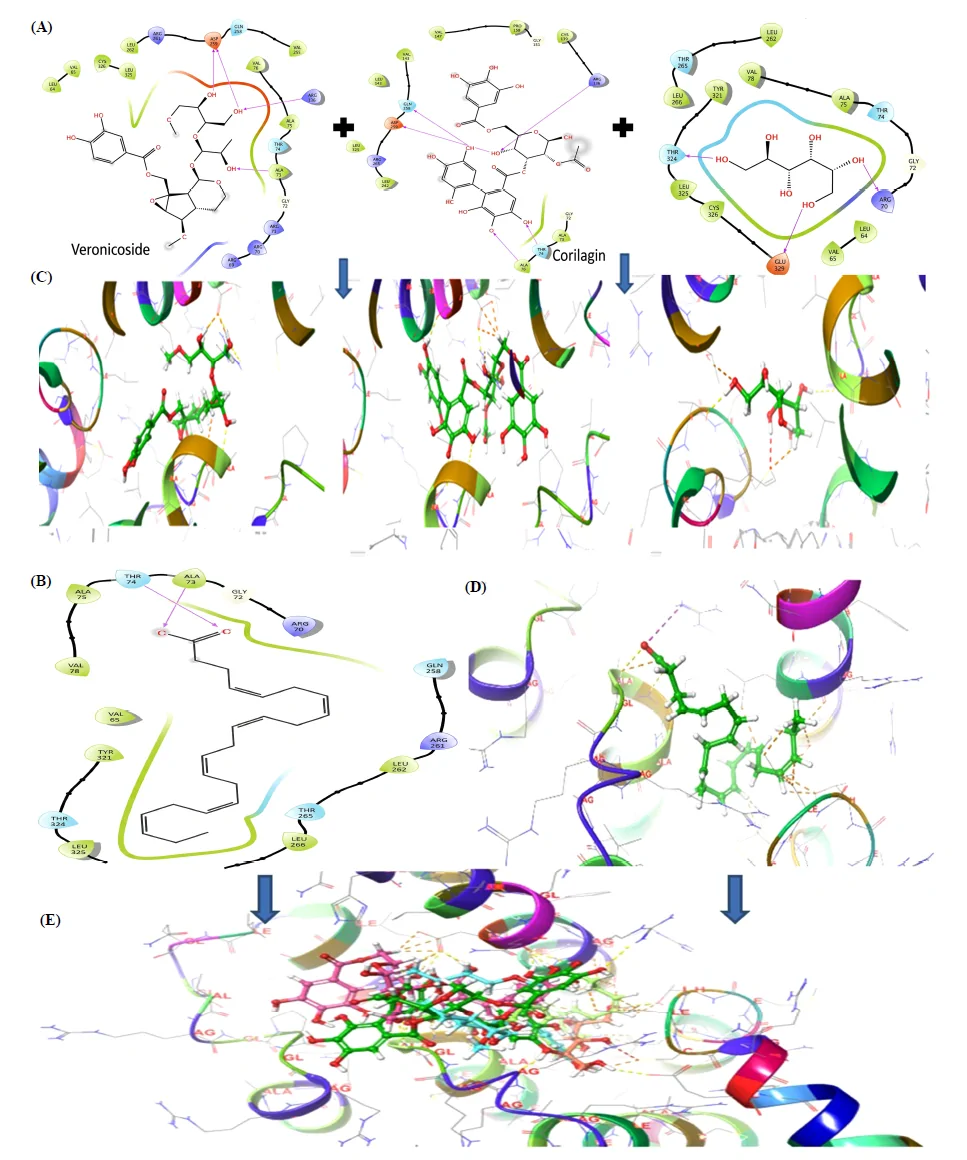
(**A**−**C**) Binding interaction of compound Veronicoside, corilagin, and Malic acid against GPR40 (PDB ID; 4PHU). (**B**−**D**) Binding interaction of standard drug DHA against GPR40. (**E**) Superimposition of compound Veronicoside, corilagin, and Malic acid (green) with DHA (brown) against GPR40.

**Table 1:** Quantification of Major Phytoconstituents in Plant Extracts.

**Phytoconstituent**	**Concentration (% w/w)**	**RF Value**	**Linearity Range (ng/spot)**	**R^2^ Value**	**LOD (ng)**	**LOQ (ng)**	**Regression Equation**	**Content (µg/100 mg) (Mean ± SD) (n = 3)**	**% Recovery**
Cinnamic acid	3.12 ± 0.15	0.616 ± 0.039	50-600	0.981	0.58	1.76	y = 0.0037x + 0.0055	312.0 ± 15.5	98.5 ± 1.2
Ferulic acid	2.75 ± 0.12	0.531 ± 0.041	50-600	0.992	0.62	1.86	y = 0.0041x + 0.0078	275.0 ± 12.0	99.1 ± 1.5
Corilagin	3.50 ± 0.18	0.672 ± 0.037	50-600	0.987	0.65	1.95	y = 0.0052x + 0.0093	350.0 ± 18.5	97.8 ± 1.8
Caffeic acid	1.85 ± 0.10	0.489 ± 0.042	50-600	0.984	0.6	1.8	y = 0.0039x + 0.0067	185.0 ± 10.2	98.2 ± 1.4
Veronicoside	2.30 ± 0.13	0.703 ± 0.038	50-600	0.988	0.64	1.92	y = 0.0045x + 0.0030	230.0 ± 13.0	98.9 ± 1.3
Malic acid	2.90 ± 0.14	0.582 ± 0.036	50-600	0.985	0.63	1.89	y = 0.0042x + 0.0043	290.0 ± 14.0	98.7 ± 1.6

**Table 2:** Detailed binding Interaction of best-docked pose of chemical constituents against GPR120 & GPR40 based on amino acid interaction.

**S.No.**	**Plants**	**Chemical constituents**	**Docking Score**	**Targeting site**	**Interacting amino acid residue**	**Number of Hydrogen Bond**	**Hydrophobic Interaction**
1.	DHA	Std. DHA *(Docosahexaenoic acid)*	-8.915	GPR120	Arg-2258 Typ-91 Arg-183 Tyr-2240	4	6
	DHA		-13.39	GPR40	Ala- 73 Thr-74	3	7
2.	Katuki *(Picrorhiza kurroa)*	Cinnamic acid	-7.580	GPR 120	Tyr-2240 Tyr-91 Arg-2258 Arg-183	2	4
		Veronicoside	-10.49	GPR 40	Ala-73 Asp-259 Arg-73	5	3
3.	Bhumyamlaki *(Phyllanthus niruri)*	Corilagin	-7.857	GPR 120	Arg-136 Asp-259 Thr-74 Ala-75	4	5
		Caffeic acid	-9.991	GPR 40	Arg-2258 Arg-183 Tyr-2240 Tyr-91	4	6
4.	Guduchi *(Tinospora cordifolia)*	Malic acid	-6.744	GPR 120	Arg-70 Glu-329 Thr-324	3	4
		Ferulic acid	-10.68	GPR 40	Arg-2258 Arg-183 Tyr-91 Tyr-2240	3	5

**Table 3:** *In silico* pharmacokinetic analysis of selective phytoconstituents from *T. cordifolia, P. niruri, and P. kurroa.*

**Herbs**	**Phytoconstituents**	**Predicted Oral Bioavailability**	**Predicted Drug-Likeness¥**	**Pharmacokinetics**				
				**CYP£**	**P-gp Substrate**	**BBB Permeability**	**GI Absorption**	**Log Kp (cm/s)**
*Tinospora cordifolia*	Malic acid	0.56/ Yes	Yes	No	No	No	High	-8.01
	Ferulic acid	0.85/Yes	Yes	No	No	No	High	-6.02
*Phyllanthus niruri*	Corilagin	0.85/Yes	Yes	No	No	Yes	High	-6.08
	Caffeic acid	0.56/Yes	Yes	No	No	No	High	-6.58
*Picrorhiza kurroa*	Cinnamic acid	0.85/Yes	Yes	No	No	Yes	High	-5.17
	Veronicoside	0.76/Yes	Yes	No	No	No	High	-8.86

**Note: ** CYP-Cytochrome P450, are the Phase-I drug metabolising enzymes responsible for >95% xenobiotic’s metabolism; P-gp-P-glycoprotein is key efflux drug transporter; BBB-blood brain barrier plays a crucial role in prevention of drug transport into the brain; whereas, Kp explains skin permeability coefficient. Kp is linearly correlated with lipophilicity and molecular size. The more negative the log Kp (with Kp in cm/s), the less skin permeant is the molecule.

^£^ Predicted CYP Inhibitionfor CYP1A2, CYP2C9, CYP2C19, CYP2D6and CYP3A4.

^¥^ Predicted Drug-likeness (Results of Lipinski, Ghose, Veber, Egan and Muegge rules).

**Table 4:** MM/GBSA studies of the six selective phytoconstituents from *T. cordifolia, P. niruri,* and *P. kurroa* against GPR120 & GPR40.

**S. No.**	**Phytoconstituents**	**Targets**	**E_vdw_d**	**E_coul_e**	**ΔG_bind_**
1.	DHA (Docosahexaenoic acid)	GPR120	-0.8	-8.79	-38.545
		GPR40	-0.5	-8.63	-41.252
2	Malic acid	GPR 120	-0.4	-3.1	-34.0898
3	Veronicoside		-4.88	-3.1	-41.0839
4	Corilagin		-2.2	-28.4	-41.343
5	Caffeic acid	GPR 40	-0.2	-8.6	-50.344
6	Ferulic acid		-1.2	-9.4	-44.3965
7	Cinnamic acid		-0.4	-7.1	-48.9339

**Abbreviation:** ^d^Van der Waals energy. ^e^Coulomb energy.

## Data Availability

The authors confirm that the data supporting the findings of this research are available within the article.

## LIST OF ABBREVIATIONS

T2DM: Type 2 Diabetes Mellitus
NAFLD: Non-Alcoholic Fatty Liver Disease
GPCRs: G-protein-coupled Receptors
FFARs: Free Fatty Acid Receptors
CCK: Cholecystokinin
MS: Mass Spectrometric
