# Supplementary material for: In Silico Exploration of Ayurvedic Extract for Targeting Diabetes Mellitus and Non-alcoholic Fatty Liver Disease via GPR120 and GPR40
Source: Curr Diabetes Rev. 2025 Jun 12;22(6):E15733998380420. doi: 10.2174/0115733998380420250411073427 (PMC13714745; doi:10.2174/0115733998380420250411073427)
Supplement: Supplementary file 1 [file CDR-22-6-E15733998380420_SD1.pdf]

Supplementary Material

In Silico Exploration of Ayurvedic Extract for Targeting Diabetes Mellitus and Non-alcoholic Fatty Liver Disease *via* GPR120 and GPR40

Priyanka Sharma[1], Kalicharan Sharma[2], Anoop Kumar[1] and Mukesh Nandave[1,*]

[1]*Department of Pharmacology, Delhi Pharmaceutical Sciences and Research University, New Delhi, 110017, India;* [2]*Department of Pharmaceutical Chemistry, ISF College of Pharmacy, Moga, Punjab, 142001, India*

Chemical and physiochemical parameter of raw plants *Tinospora cordifolia*, *Phyllanthus niruri* and *Picrorhiza kurroa.*

S. No.	Chemical and Physiochemical Parameter	Plants	Results %
1	Passport data of plant material (place and date of collection)<br>Part of plant used, botanical description,	Attached	
2	Acid Insoluble Ash	a.<br>b.<br>c.	40.55<br>4.5<br>10
3	Ash Value	a.<br>b.<br>c.	0.09<br>0.12<br>0.2
4	Moisture Content	a.<br>b.<br>c.	4<br>12<br>6
5	Total Ash	a.<br>b.<br>c.	14<br>16<br>12
6	Alcohol extraction	a.<br>b.<br>c.	25<br>23<br>25
7	Water extraction	a.<br>b.<br>c.	48<br>45<br>46

8	Heavy metals<br><br>Lead not more than 10 ppm (Limits as per ASU Pharmacopoeia)	a.<br><br>b.<br><br>c	Complies<br><br>Complies<br><br>Complies

a. **Tinospora cordifolia*, b. *Phyllanthus niruri* and c. *Picrorhiza kurroa.*

Mass spectrometer fingerprint of hydro-alcoholic extract.

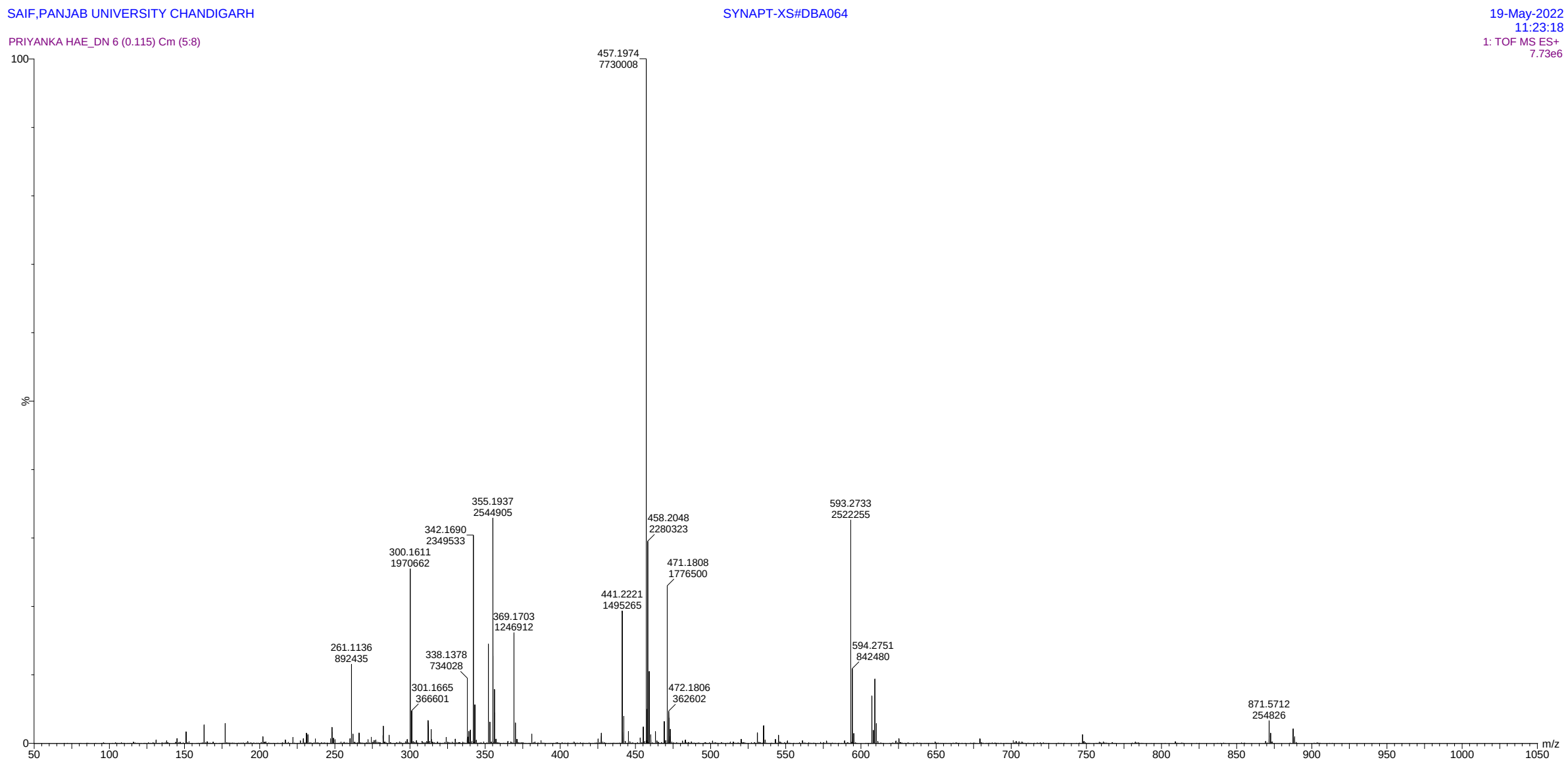

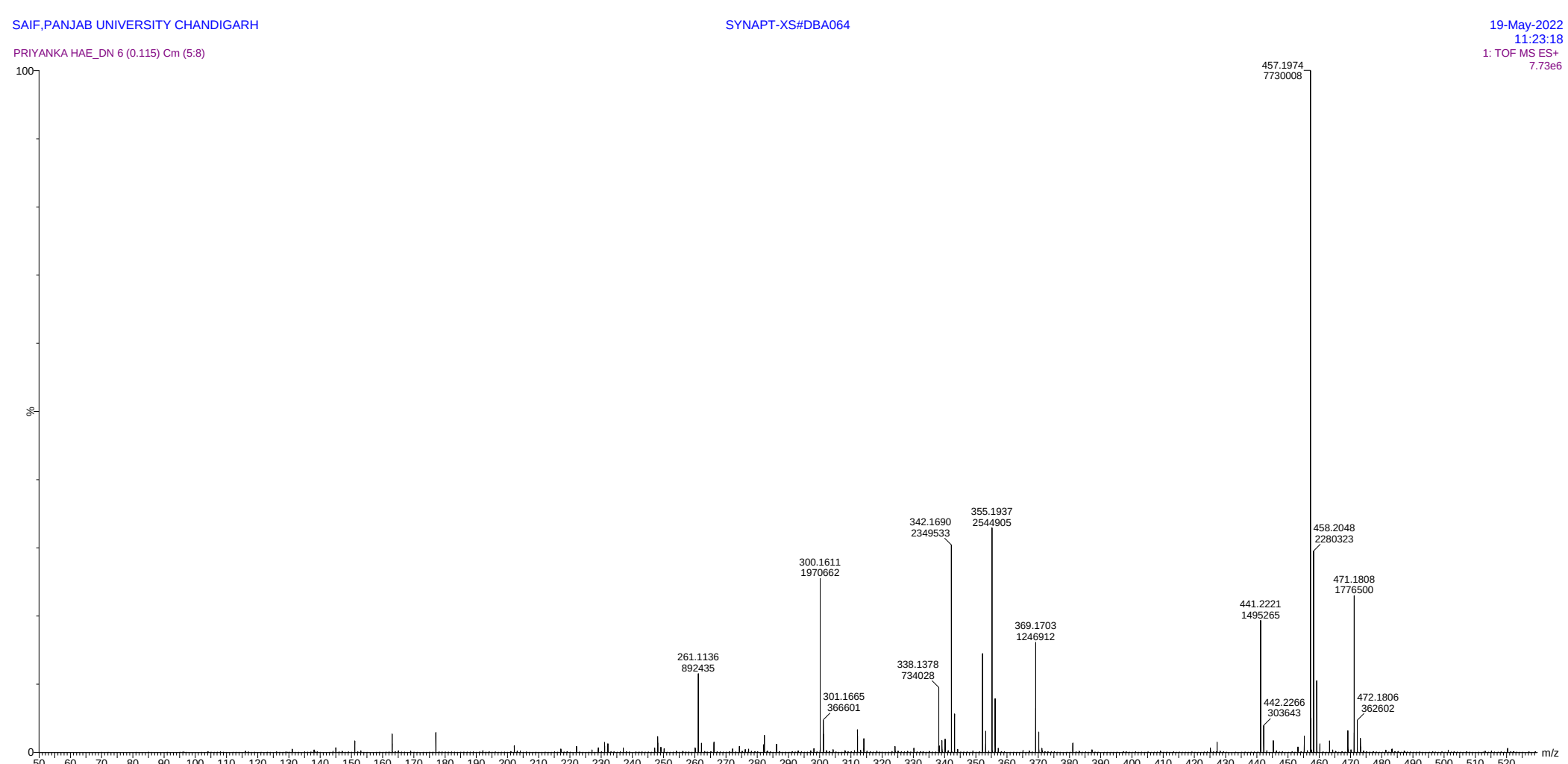

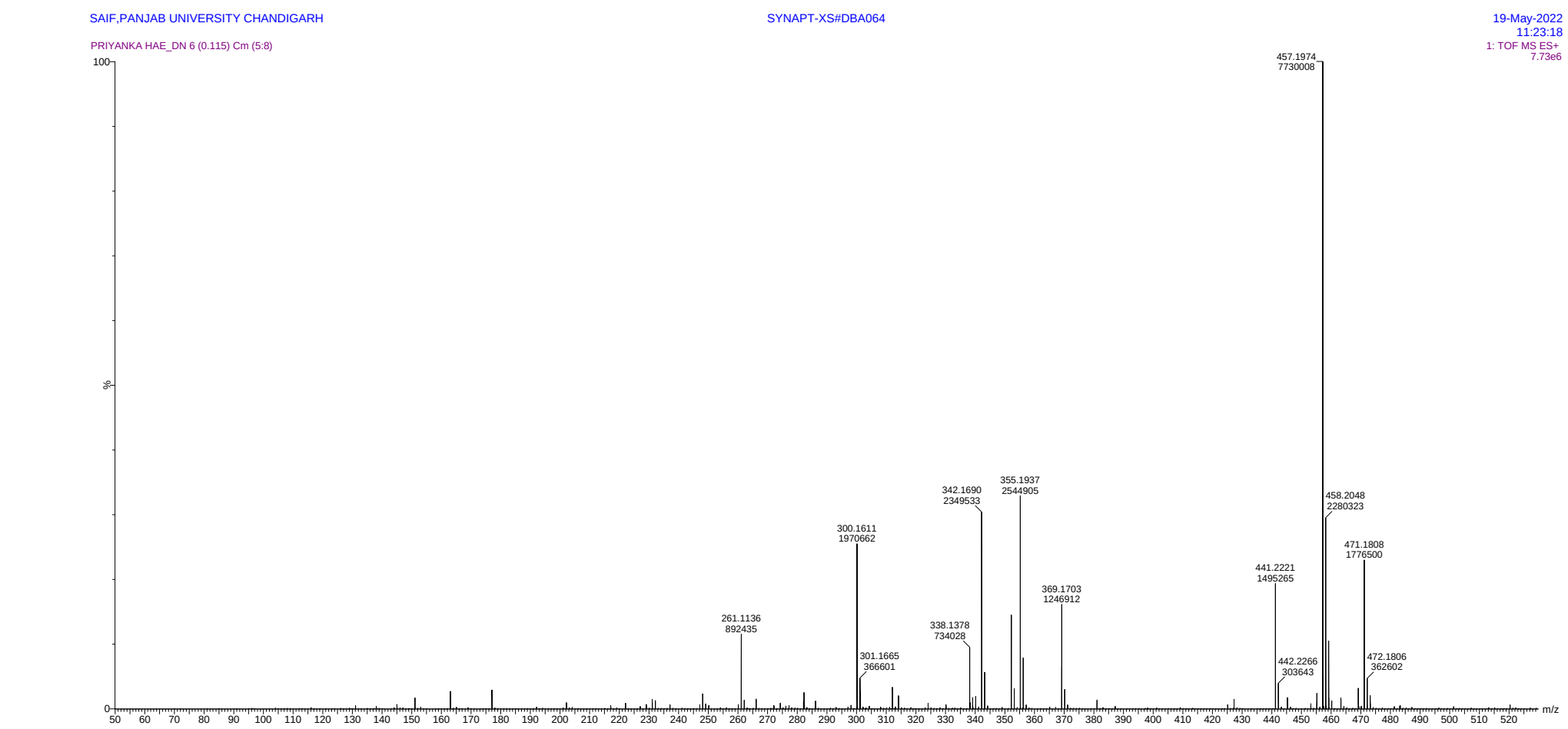
SAIF,PANJAB UNIVERSITY CHANDIGARH
SYNAPT-XS#DBA064
19-May-2022
11:23:18
PRIYANKA HAE_DN 6 (0.115) Cm (5:8)
1: TOF MS ES+
7.73e6
457.1974
7730008
458.2048
2280323
471.1808
1776500
441.2221
1495265
442.2266
303643
472.1806
362602
355.1937
2544905
342.1690
2349533
369.1703
1246912
338.1378
734028
300.1611
1970662
301.1665
366601
261.1136
892435
%
m/z

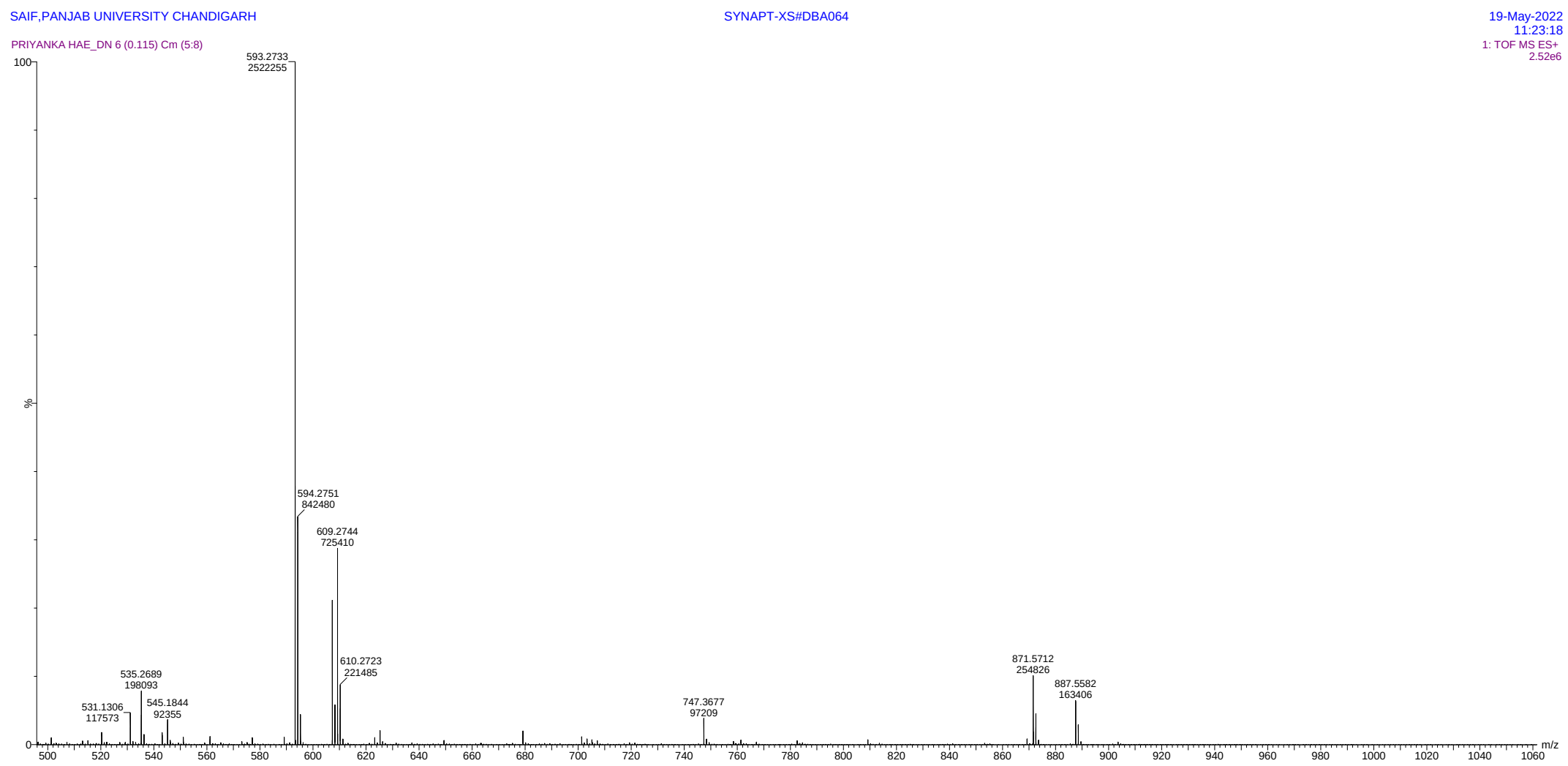
SAIF,PANJAB UNIVERSITY CHANDIGARH
SYNAPT-XS#DBA064
19-May-2022
11:23:18
PRIYANKA HAE_DN 6 (0.115) Cm (5:8)
1: TOF MS ES+
2.52e6
593.2733
2522255
594.2751
842480
609.2744
725410
610.2723
221485
535.2689
198093
531.1306
117573
545.1844
92355
747.3677
97209
871.5712
254826
887.5582
163406
%
m/z

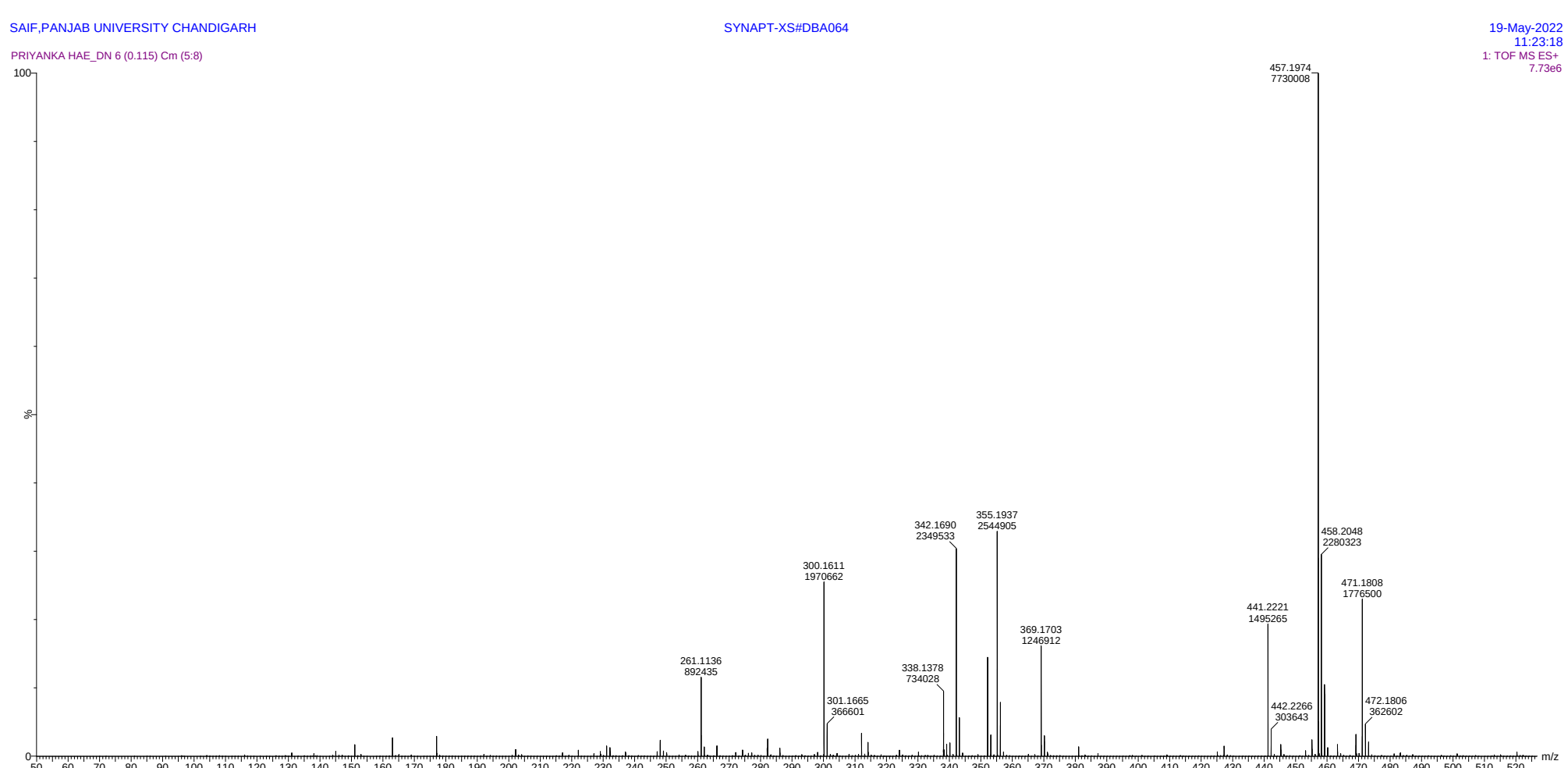
SAIF,PANJAB UNIVERSITY CHANDIGARH
SYNAPT-XS#DBA064
19-May-2022
11:23:18
PRIYANKA HAE_DN 6 (0.115) Cm (5:8)
1: TOF MS ES+
7.73e6
457.1974
7730008
458.2048
2280323
471.1808
1776500
441.2221
1495265
442.2266
303643
472.1806
362602
355.1937
2544905
342.1690
2349533
369.1703
1246912
338.1378
734028
300.1611
1970662
301.1665
366601
261.1136
892435
%
m/z

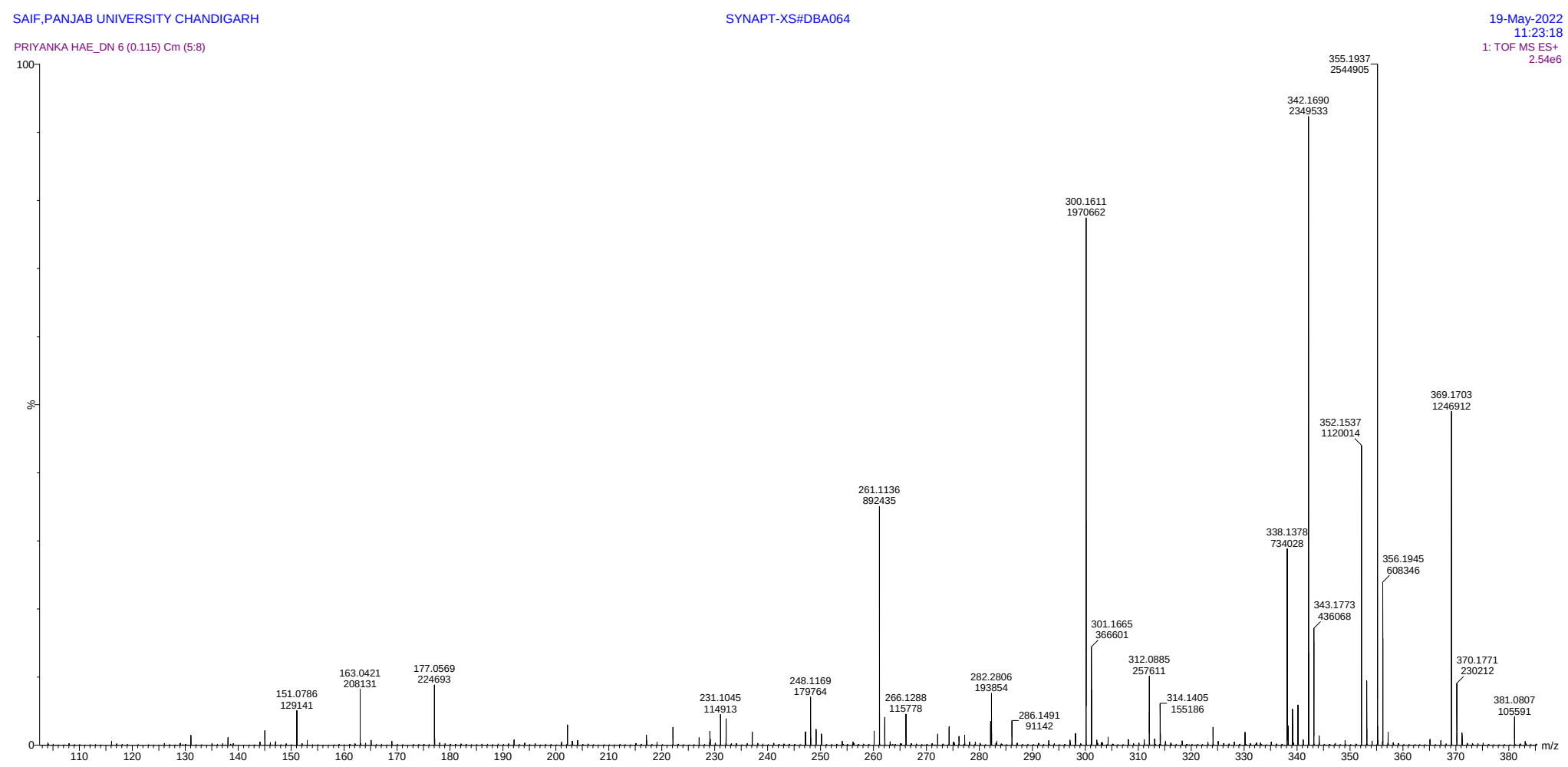
SAIF,PANJAB UNIVERSITY CHANDIGARH
SYNAPT-XS#DBA064
19-May-2022
11:23:18
PRIYANKA HAE_DN 6 (0.115) Cm (5:8)
1: TOF MS ES+
2.54e6
355.1937
2544905
342.1690
2349533
300.1611
1970662
369.1703
1246912
352.1537
1120014
261.1136
892435
338.1378
734028
356.1945
608346
343.1773
436068
301.1665
366601
370.1771
230212
312.0885
257611
177.0569
224693
163.0421
208131
248.1169
179764
282.2806
193854
151.0786
129141
231.1045
114913
266.1288
115778
314.1405
155186
381.0807
105591
286.1491
91142
m/z

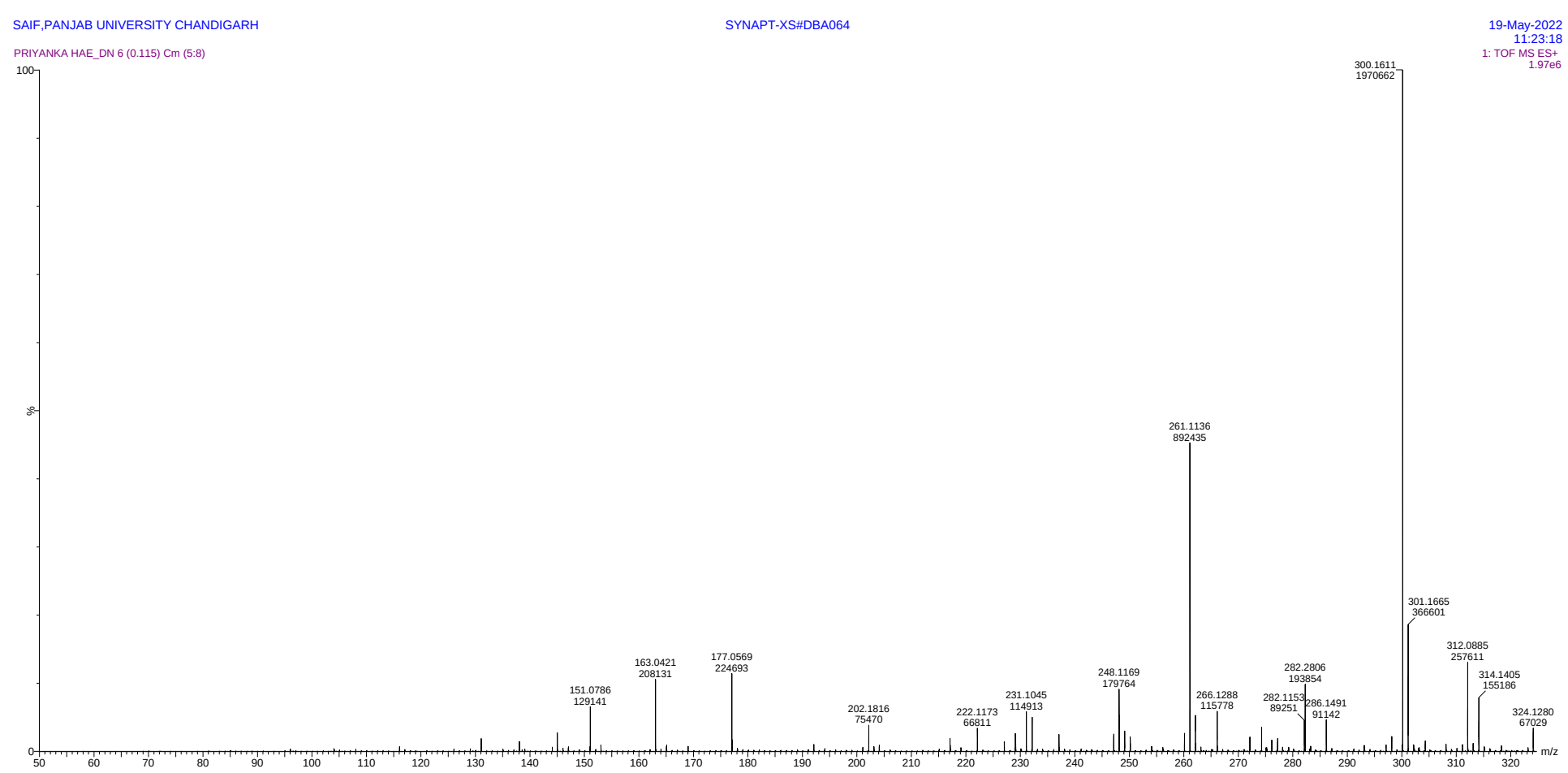
SAIF,PANJAB UNIVERSITY CHANDIGARH
SYNAPT-XS#DBA064
19-May-2022
11:23:18
PRIYANKA HAE_DN 6 (0.115) Cm (5:8)
1: TOF MS ES+
1.97e6
300.1611
1970662
261.1136
892435
301.1665
366601
312.0885
257611
163.0421
208131
177.0569
224693
151.0786
129141
202.1816
75470
222.1173
66811
231.1045
114913
248.1169
179764
266.1288
115778
282.2806
193854
282.1153
89251
286.1491
91142
314.1405
155186
324.1280
67029
m/z

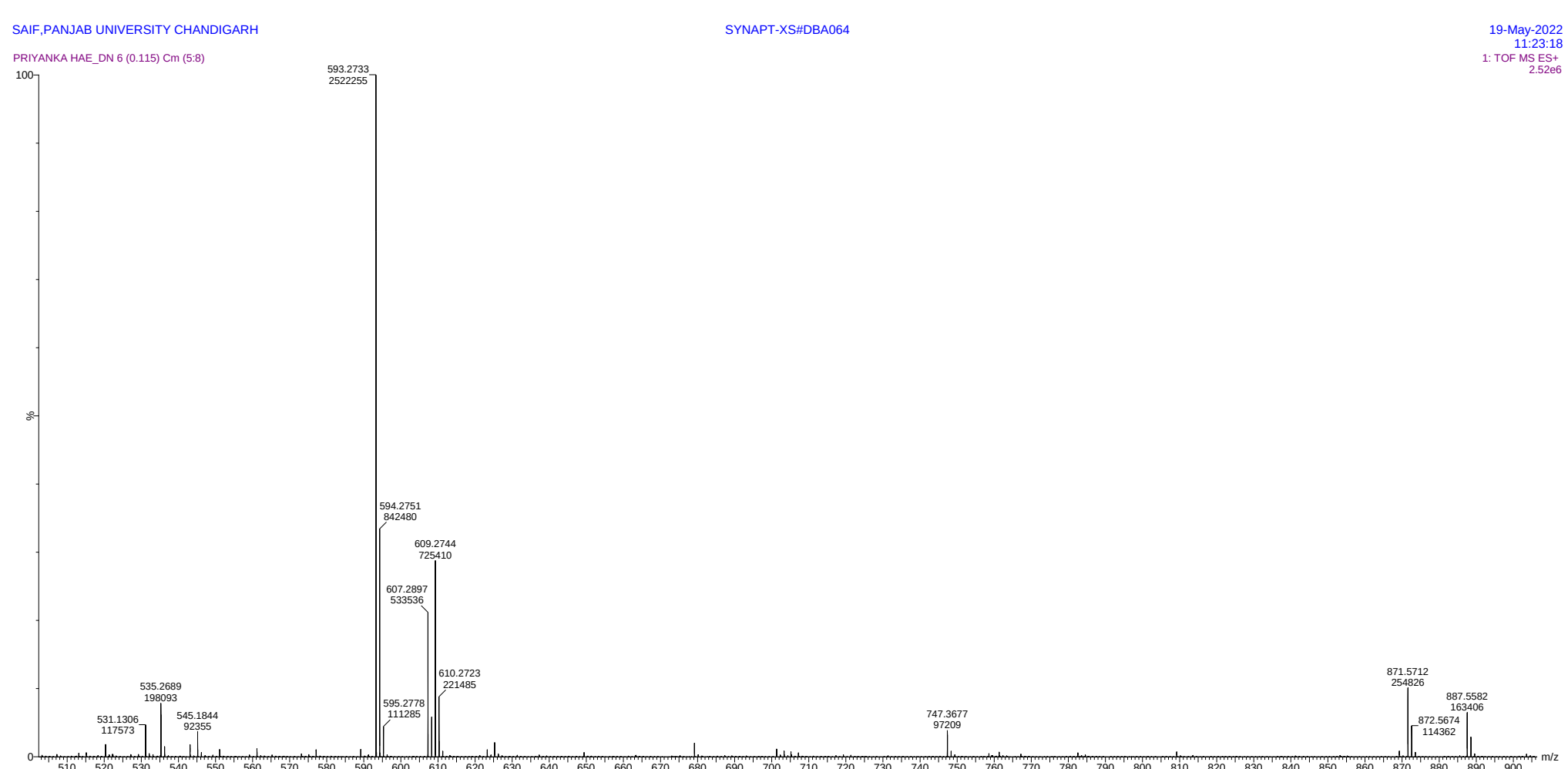
SAIF,PANJAB UNIVERSITY CHANDIGARH
SYNAPT-XS#DBA064
19-May-2022
11:23:18
PRIYANKA HAE_DN 6 (0.115) Cm (5:8)
1: TOF MS ES+
2.52e6
593.2733
2522255
594.2751
842480
609.2744
725410
607.2897
533536
610.2723
221485
595.2778
111285
535.2689
198093
531.1306
117573
545.1844
92355
747.3677
97209
871.5712
254826
887.5582
163406
872.5674
114362
m/z

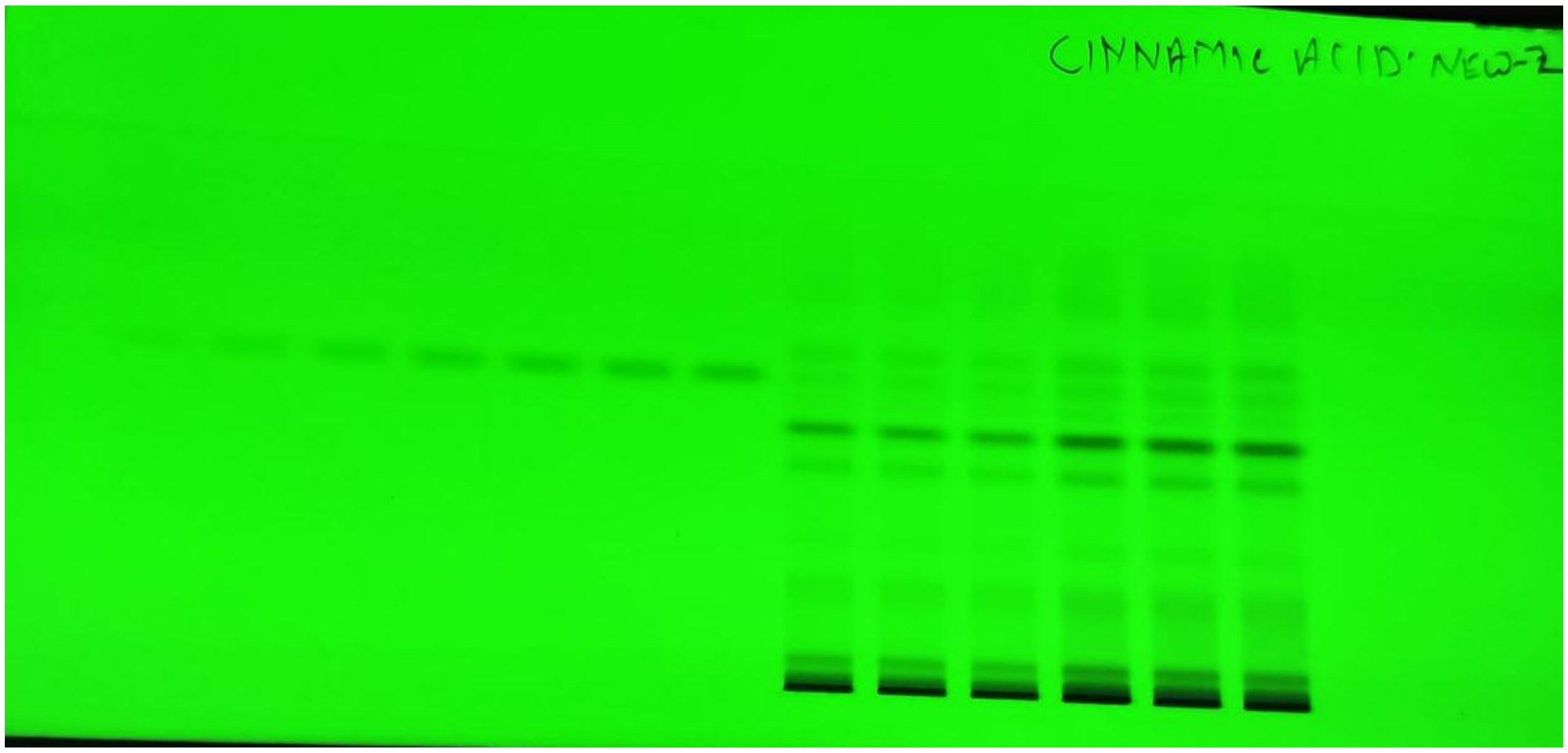
CINNAMIC ACID' NEW-2

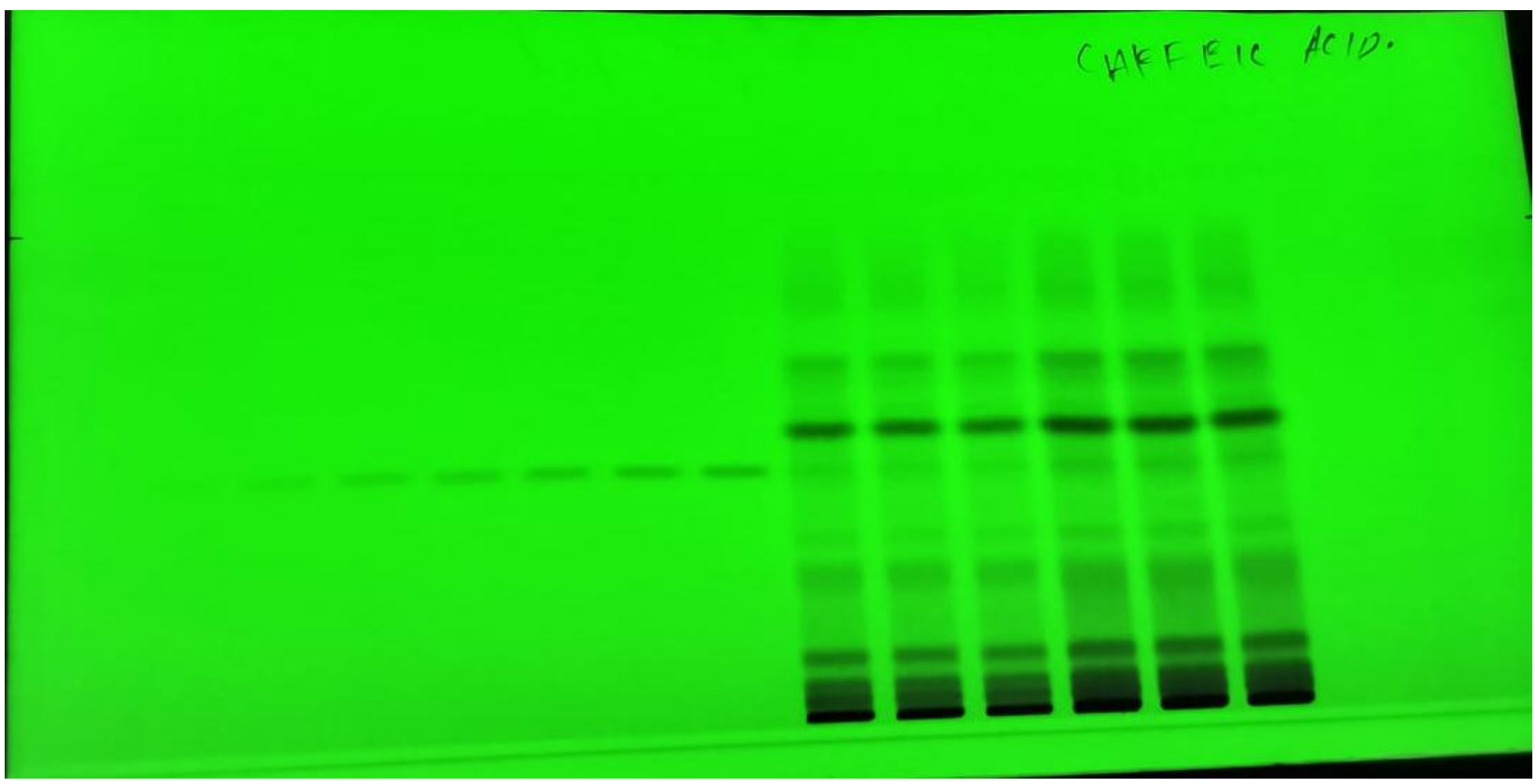
CAFFEIC ACID.

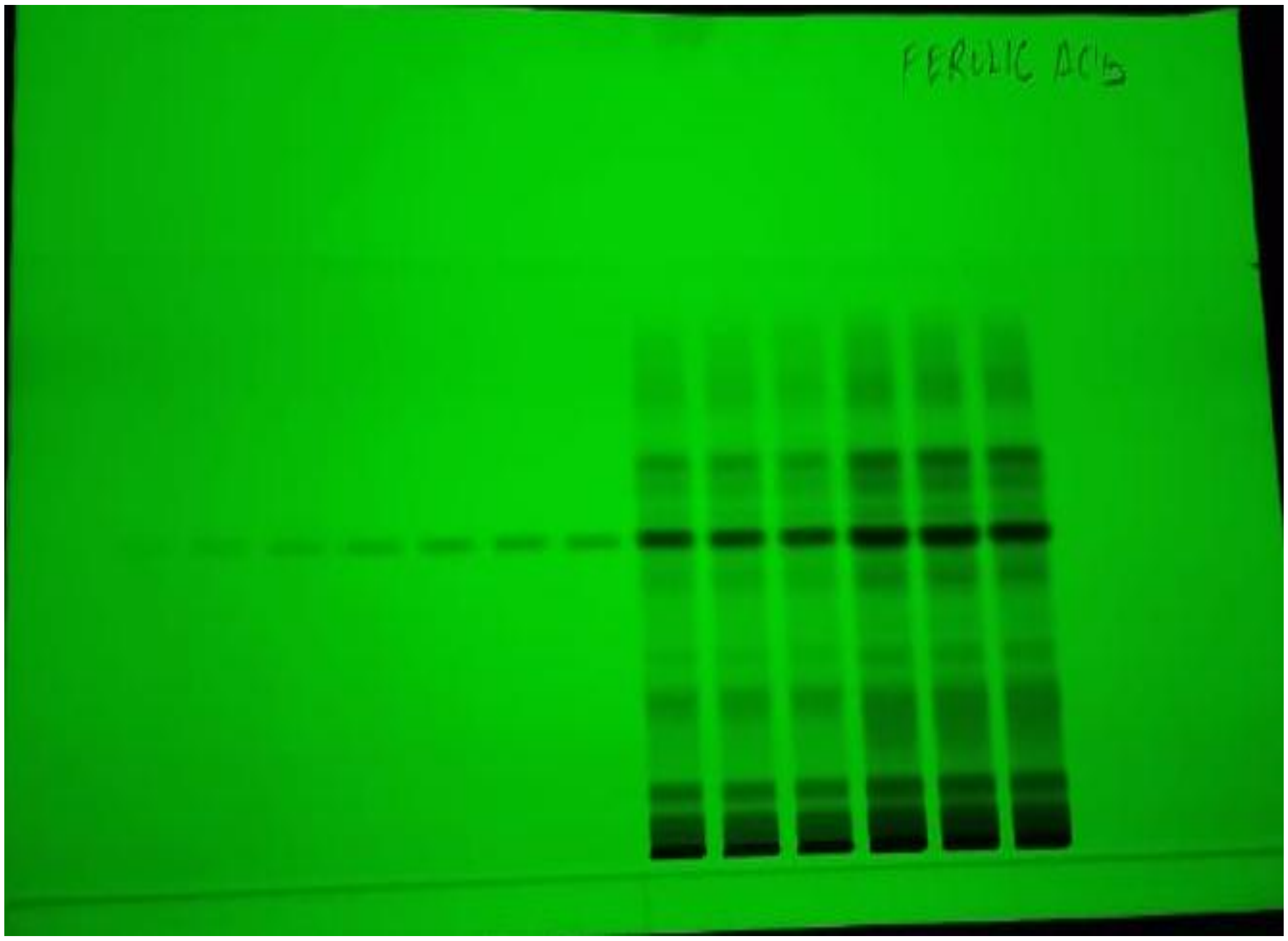

Fig. (5). HPTLC plate image under 273 nm, and 282 nm fluorescent light. (**A**) Cinnamic acid, (**B**) Caffeic acid, (**C**) Ferulic acid (**D**) All standard and sample on single plate under 316 nm.

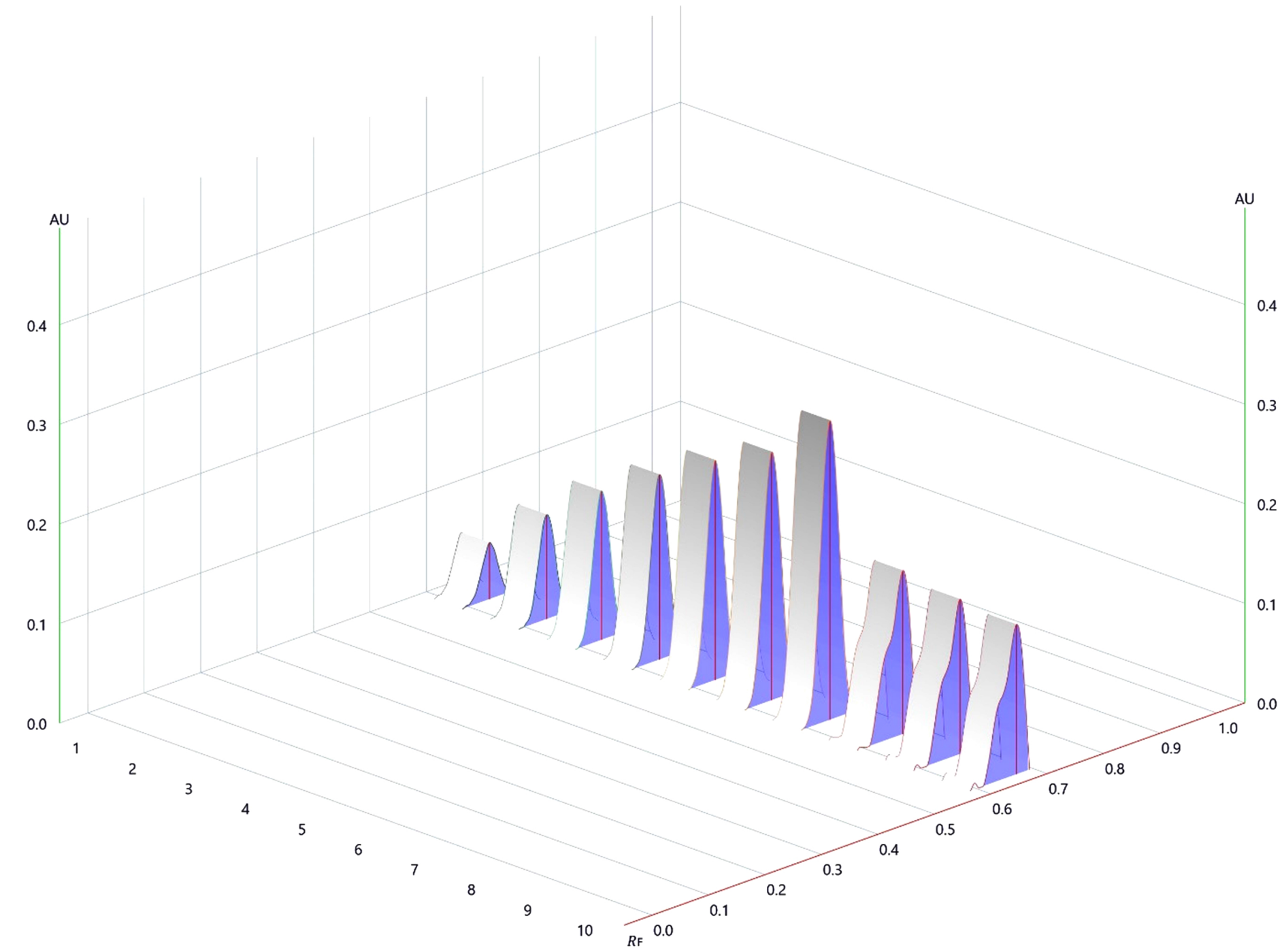
AU
0.4
0.3
0.2
0.1
0.0
1
2
3
4
5
6
7
8
9
10
RF
0.0
0.1
0.2
0.3
0.4
0.5
0.6
0.7
0.8
0.9
1.0

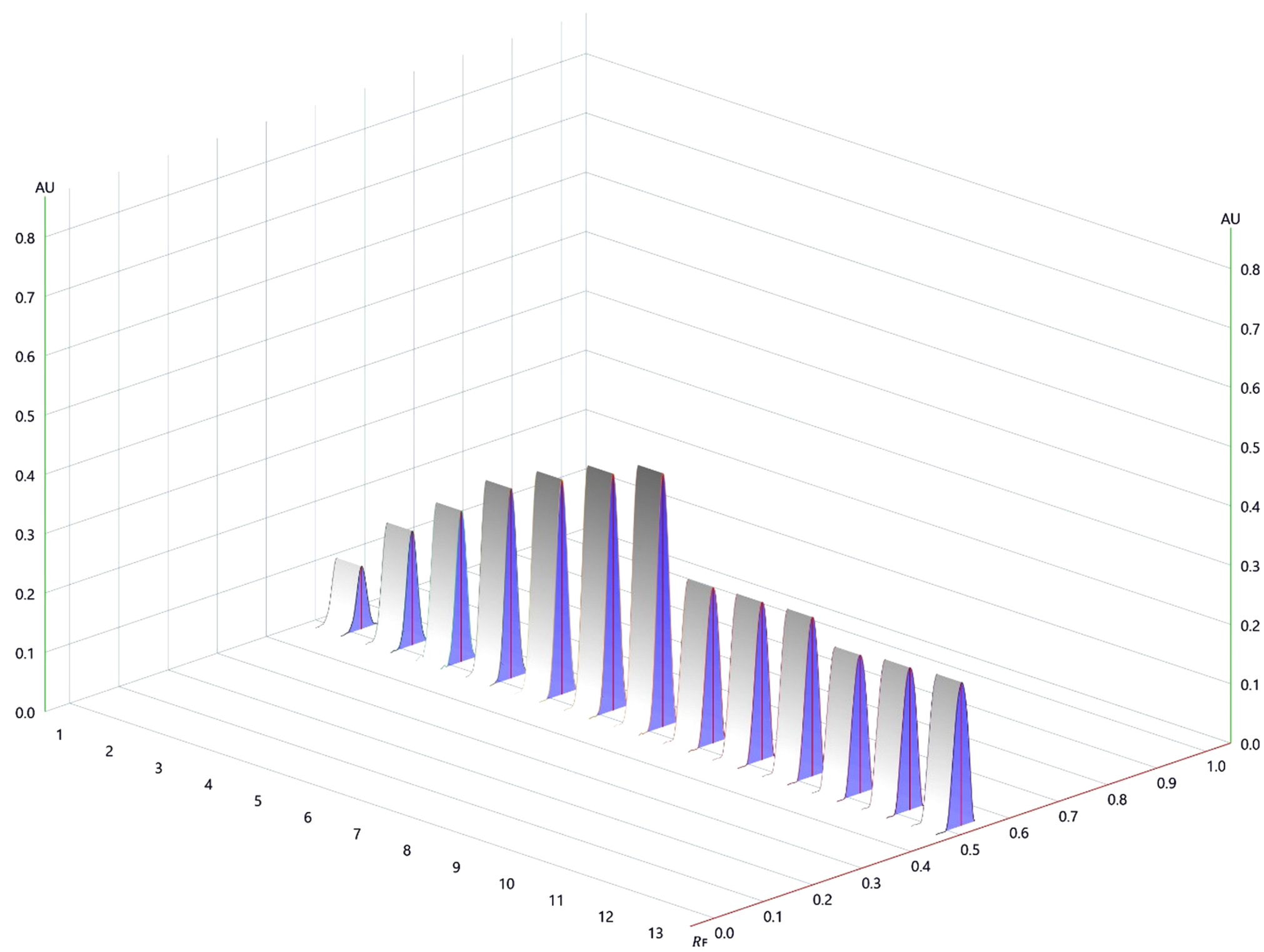
AU
0.8
0.7
0.6
0.5
0.4
0.3
0.2
0.1
0.0
1
2
3
4
5
6
7
8
9
10
11
12
13
RF
0.0
0.1
0.2
0.3
0.4
0.5
0.6
0.7
0.8
0.9
1.0

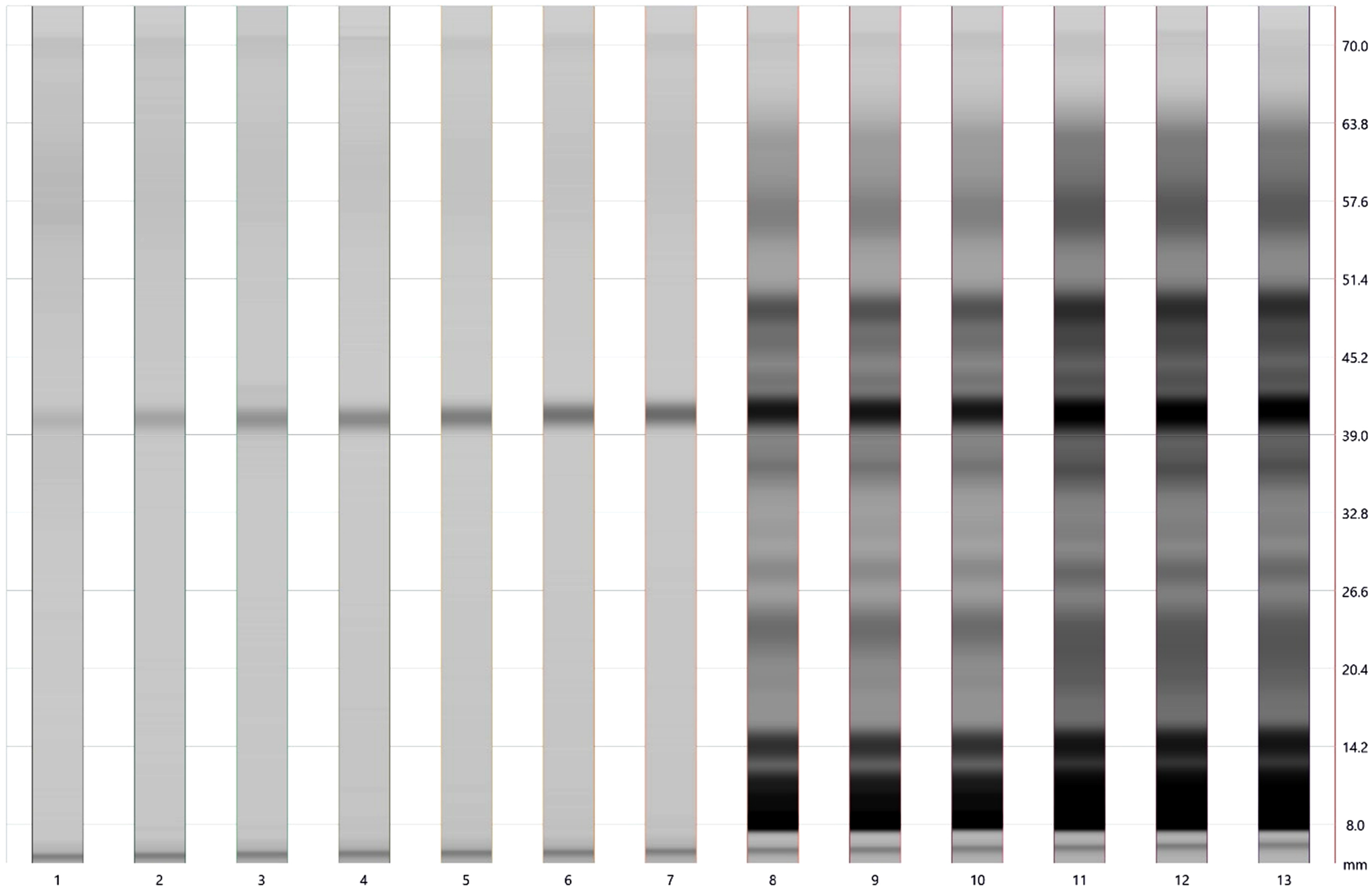
70.0
63.8
57.6
51.4
45.2
39.0
32.8
26.6
20.4
14.2
8.0
mm
1
2
3
4
5
6
7
8
9
10
11
12
13

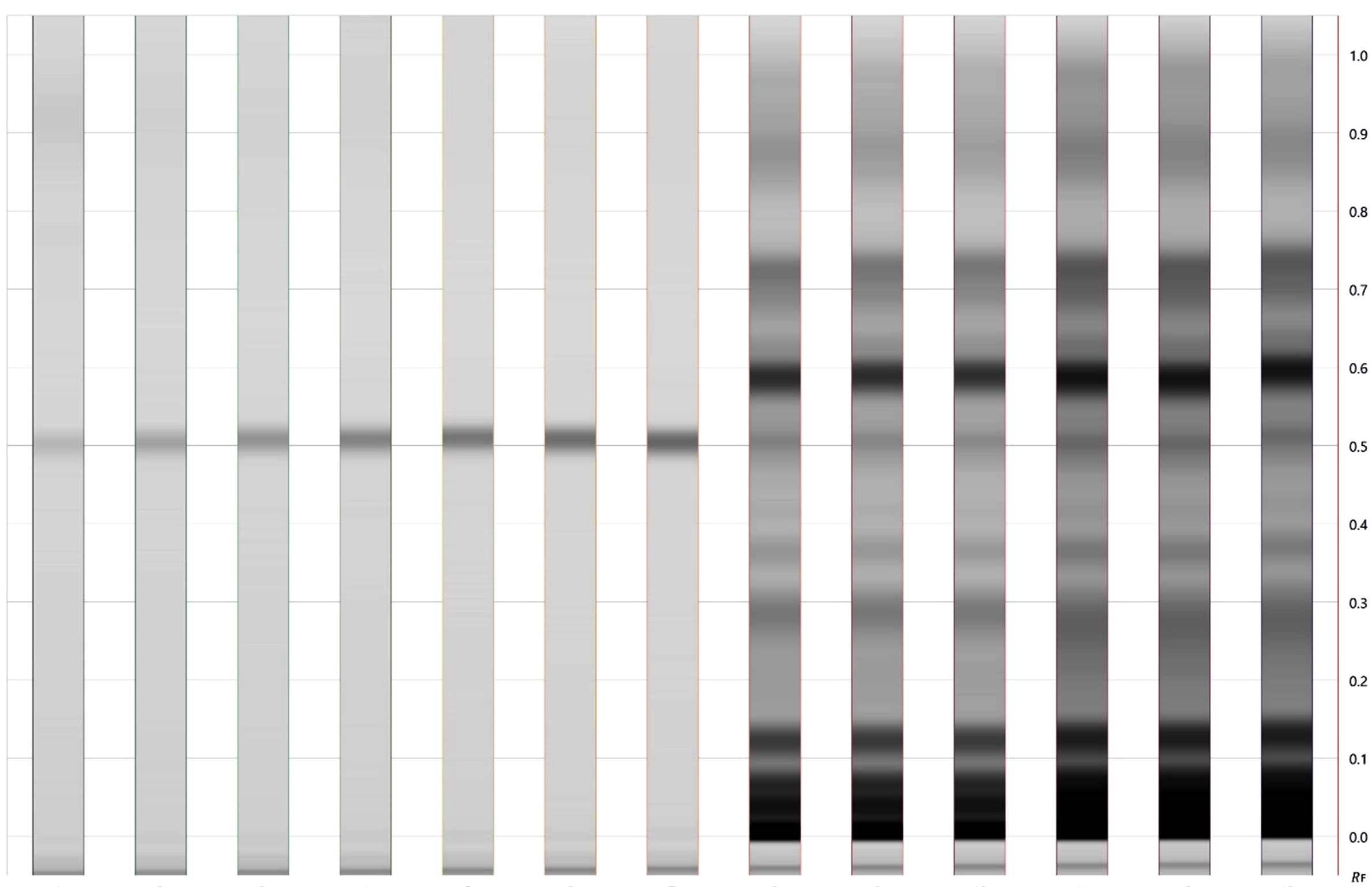
1.0
0.9
0.8
0.7
0.6
0.5
0.4
0.3
0.2
0.1
0.0
RF
1
2
3
4
5
6
7
8
9
10
11
12
13

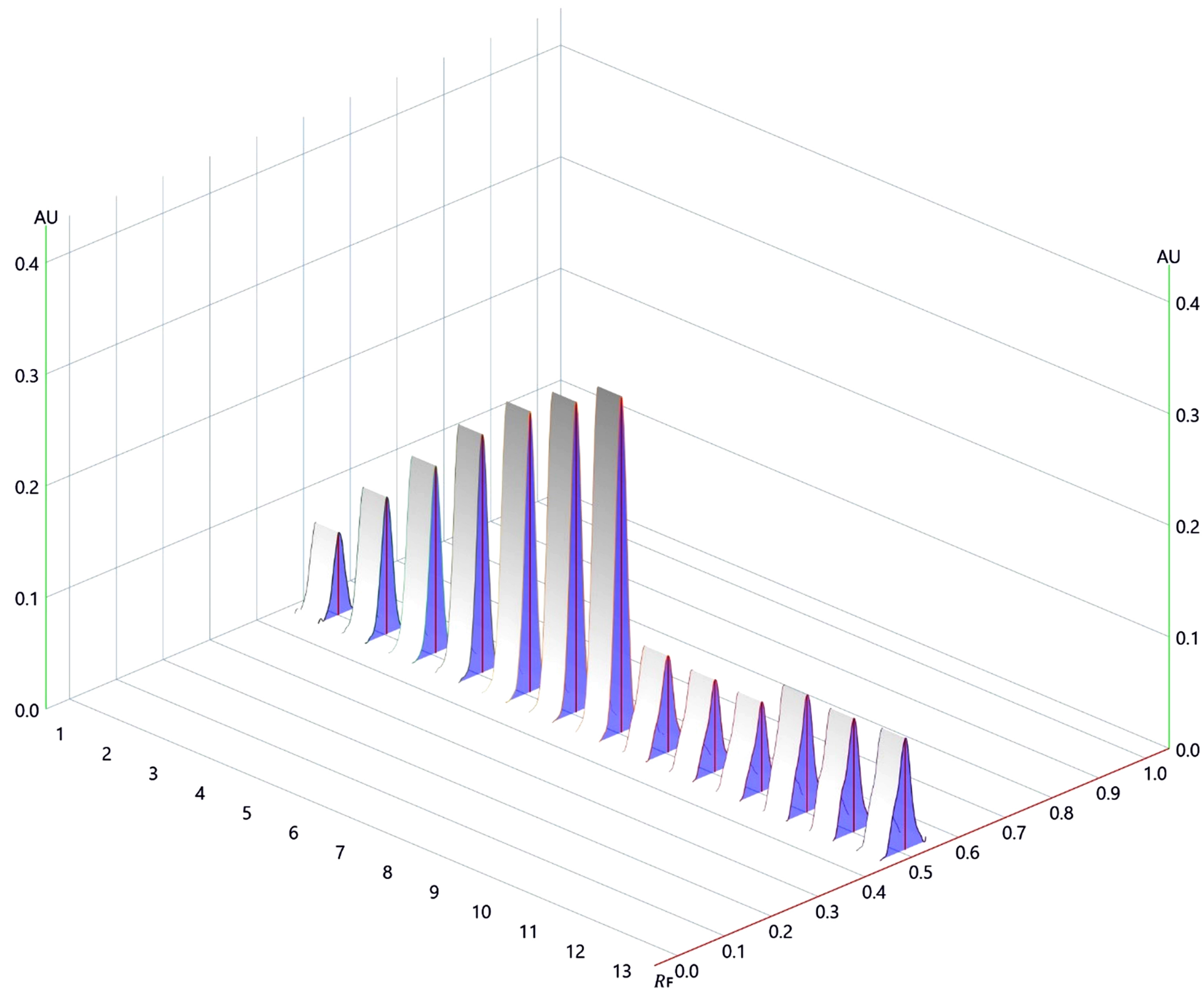
AU
0.4
0.3
0.2
0.1
0.0
AU
0.4
0.3
0.2
0.1
0.0
1
2
3
4
5
6
7
8
9
10
11
12
13
RF
0.0
0.1
0.2
0.3
0.4
0.5
0.6
0.7
0.8
0.9
1.0

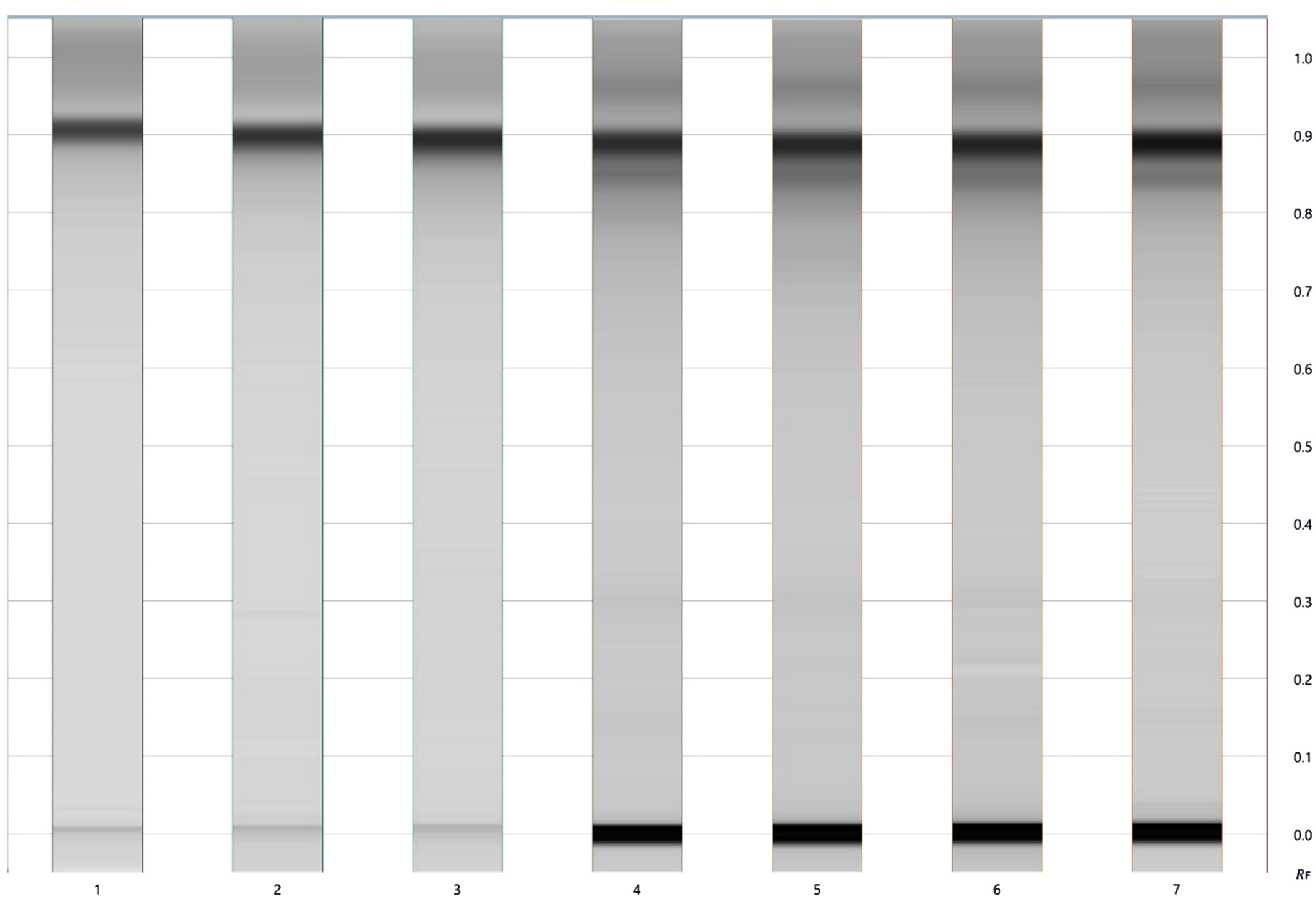
1.0
0.9
0.8
0.7
0.6
0.5
0.4
0.3
0.2
0.1
0.0
RF
1
2
3
4
5
6
7

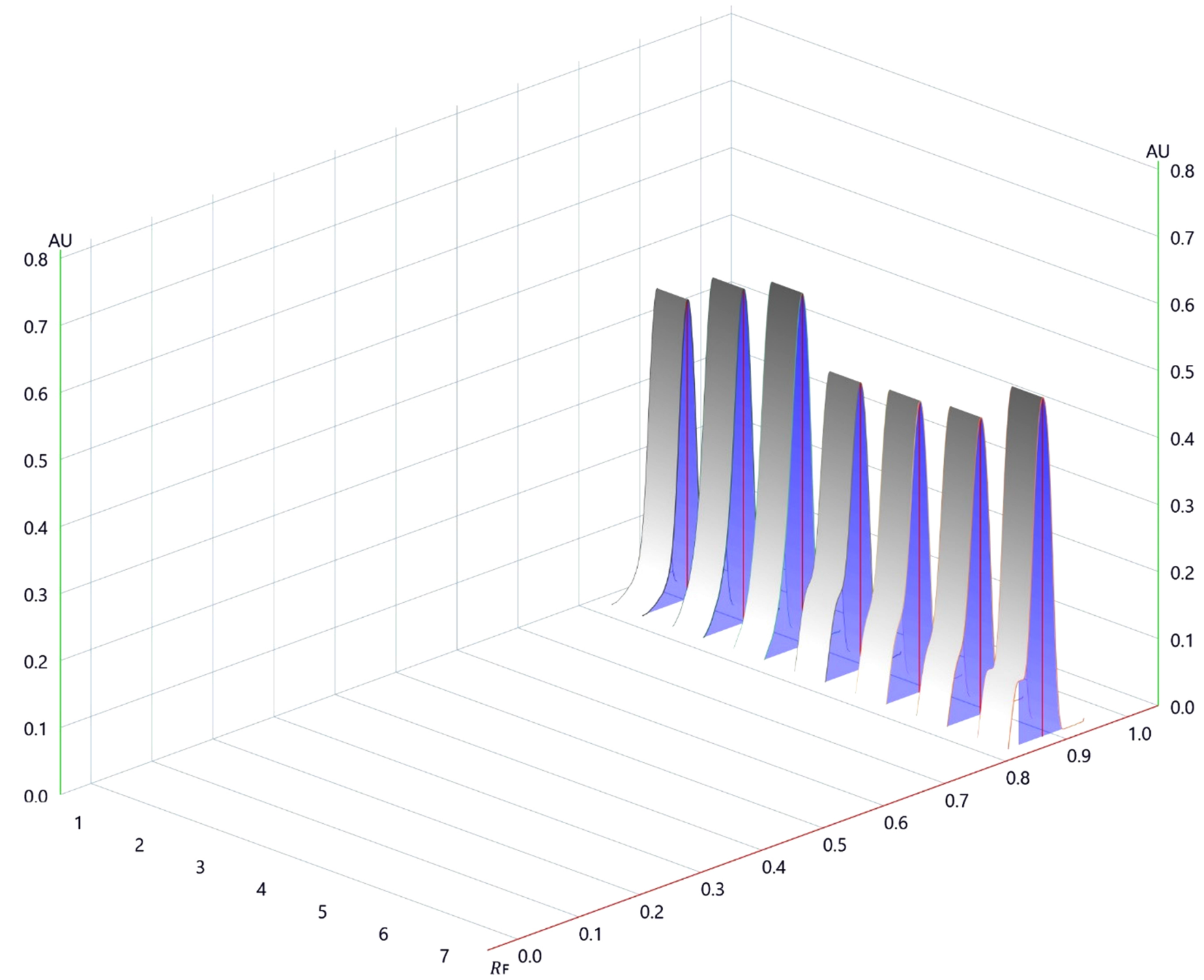
AU
0.8
0.7
0.6
0.5
0.4
0.3
0.2
0.1
0.0
1
2
3
4
5
6
7
RF
0.0
0.1
0.2
0.3
0.4
0.5
0.6
0.7
0.8
0.9
1.0

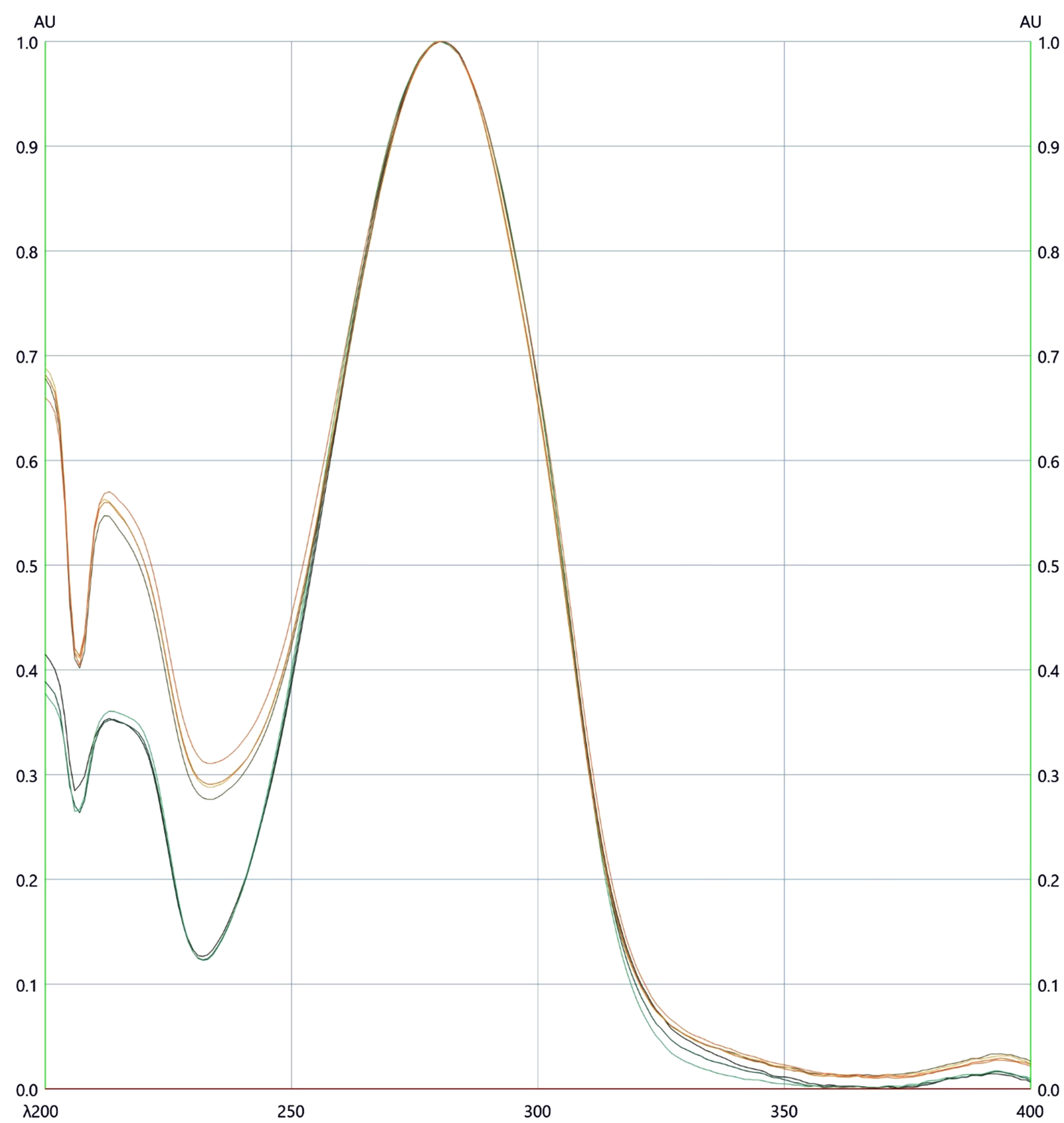
AU
1.0
0.9
0.8
0.7
0.6
0.5
0.4
0.3
0.2
0.1
0.0
λ200
250
300
350
400

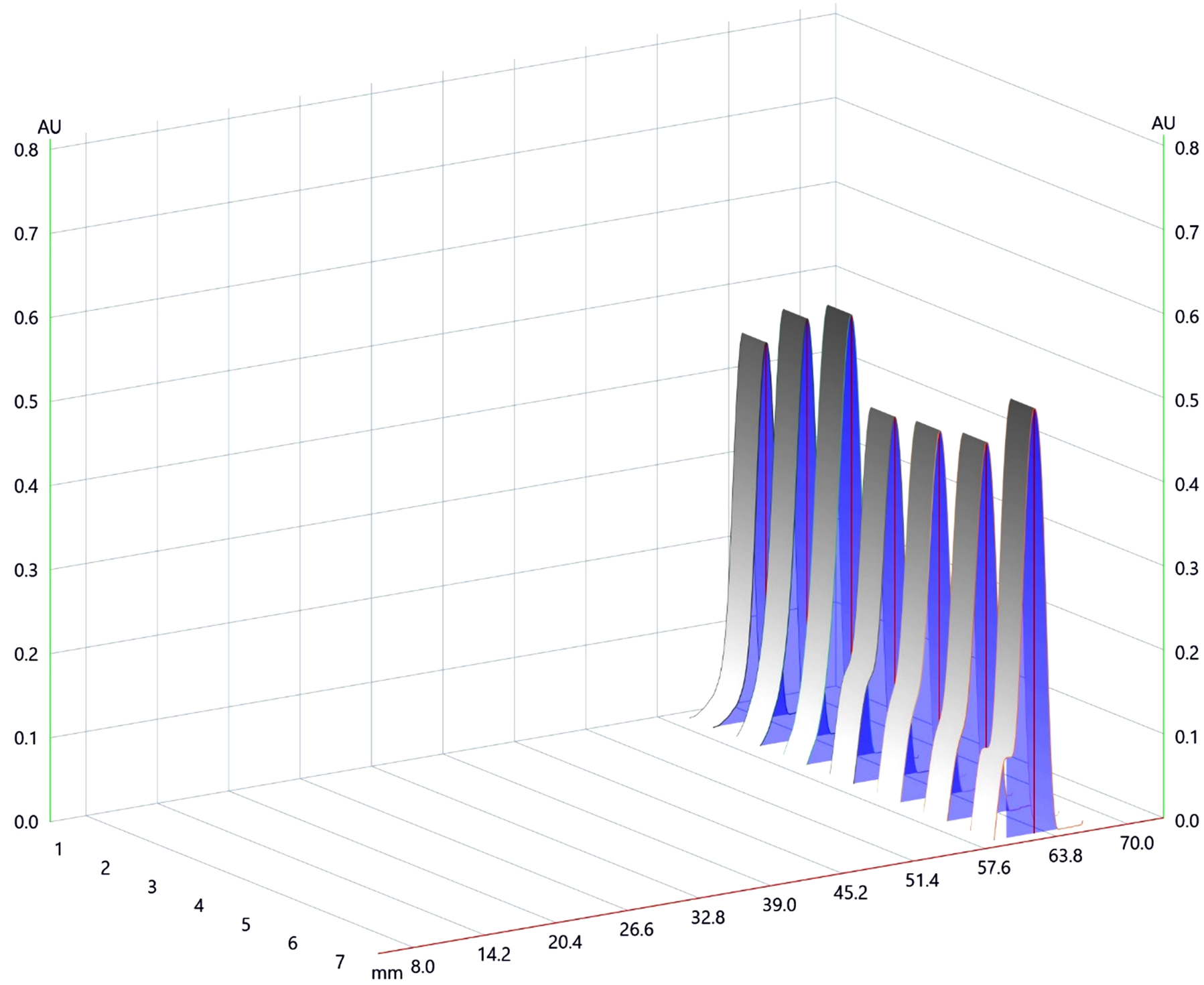
AU
0.8
0.7
0.6
0.5
0.4
0.3
0.2
0.1
0.0
1
2
3
4
5
6
7
mm
8.0
14.2
20.4
26.6
32.8
39.0
45.2
51.4
57.6
63.8
70.0

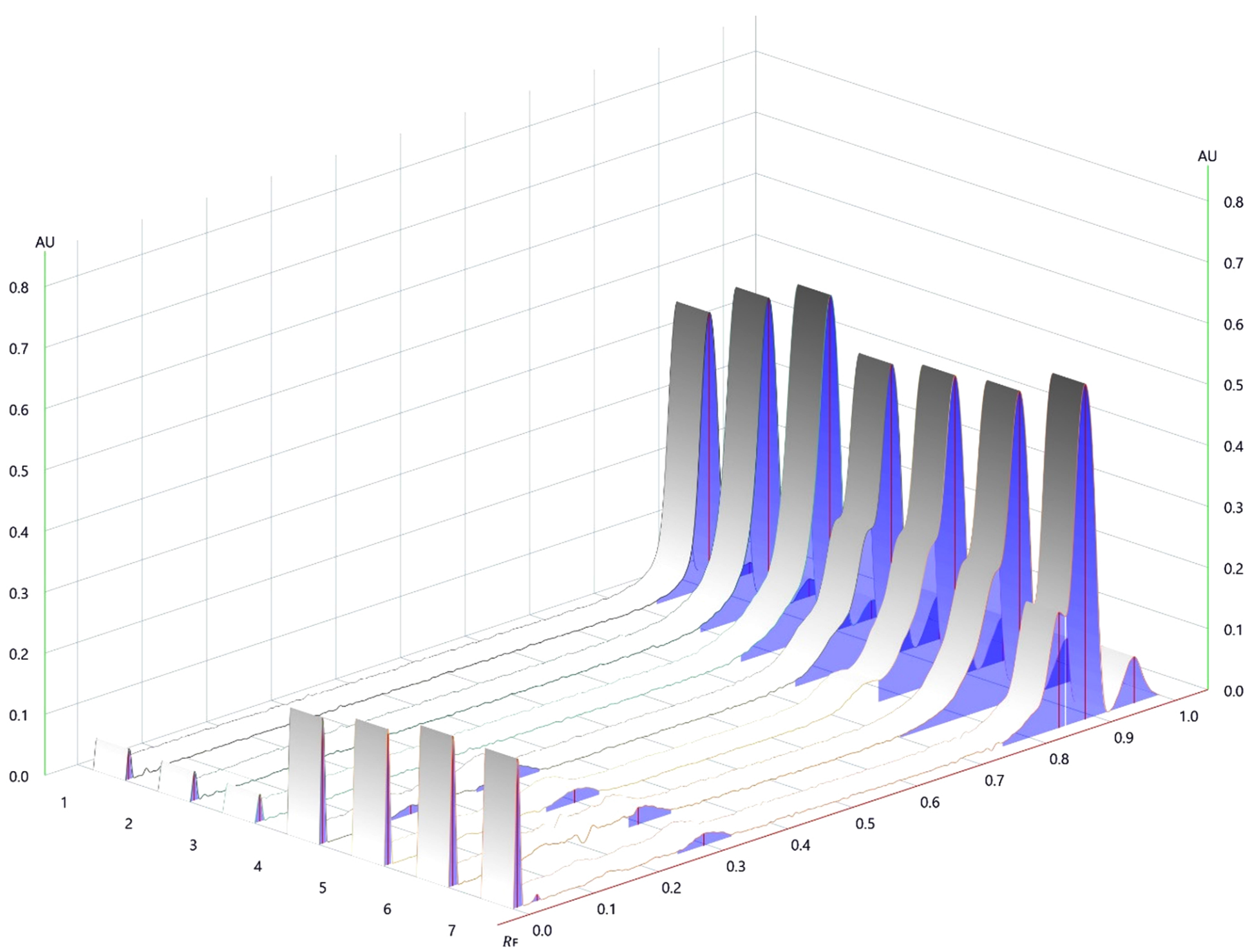
AU
0.8
0.7
0.6
0.5
0.4
0.3
0.2
0.1
0.0
1
2
3
4
5
6
7
RF
0.0
0.1
0.2
0.3
0.4
0.5
0.6
0.7
0.8
0.9
1.0

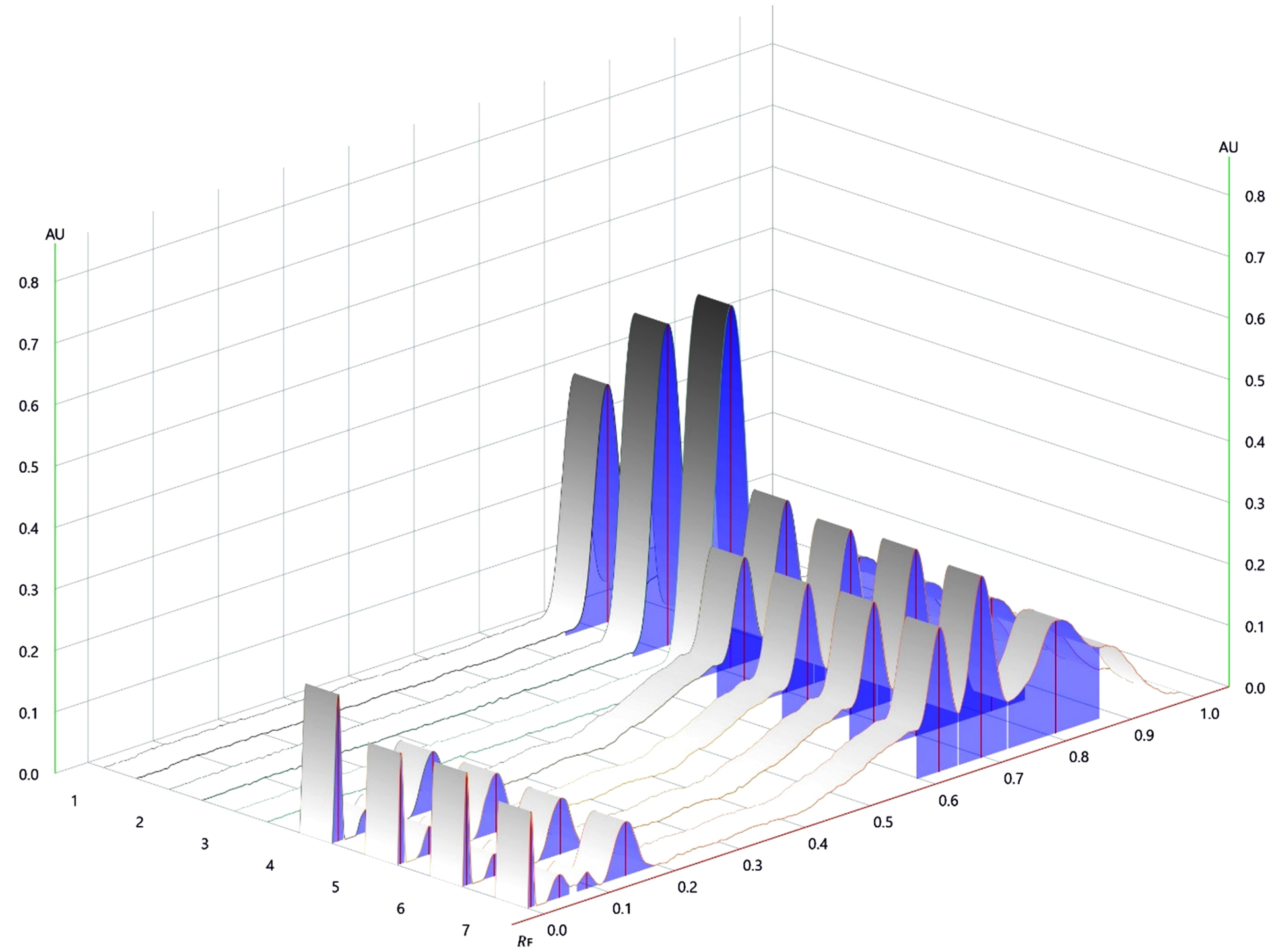
AU
0.8
0.7
0.6
0.5
0.4
0.3
0.2
0.1
0.0
1
2
3
4
5
6
7
RF
0.0
0.1
0.2
0.3
0.4
0.5
0.6
0.7
0.8
0.9
1.0

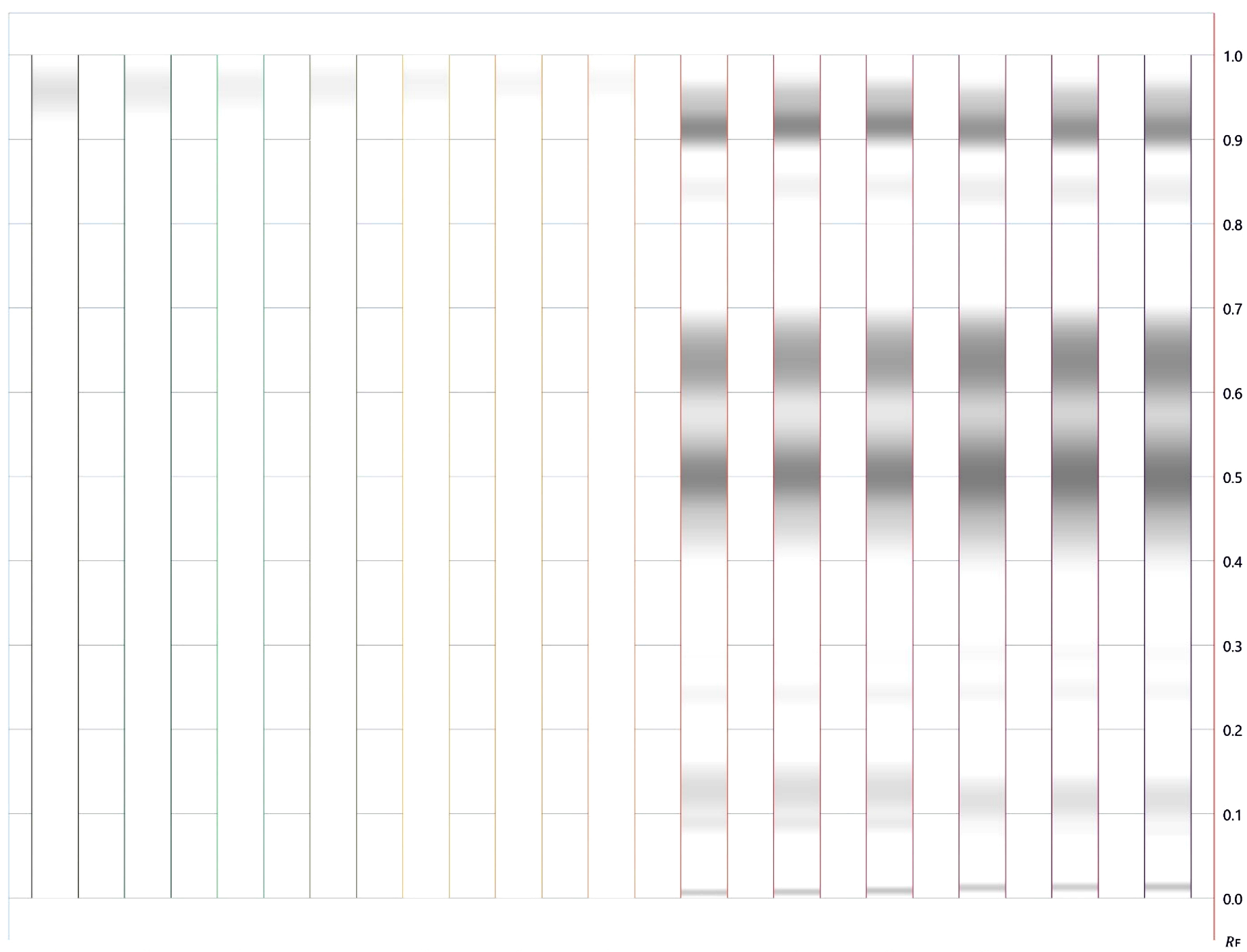
1.0
0.9
0.8
0.7
0.6
0.5
0.4
0.3
0.2
0.1
0.0
RF
1
2
3
4
5
6
7
8
9
10
11
12
13

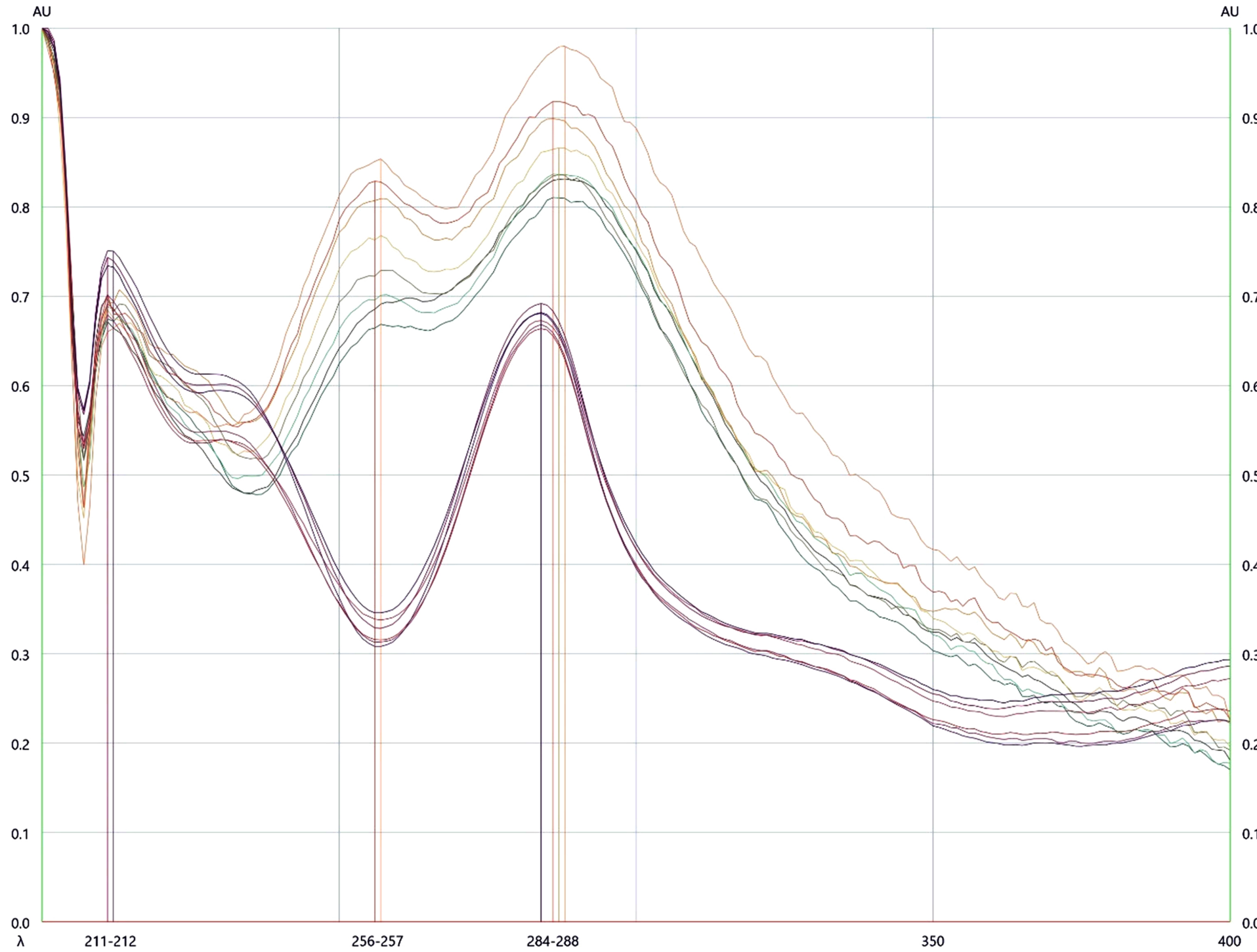
AU
1.0
0.9
0.8
0.7
0.6
0.5
0.4
0.3
0.2
0.1
0.0
λ
211-212
256-257
284-288
350
400

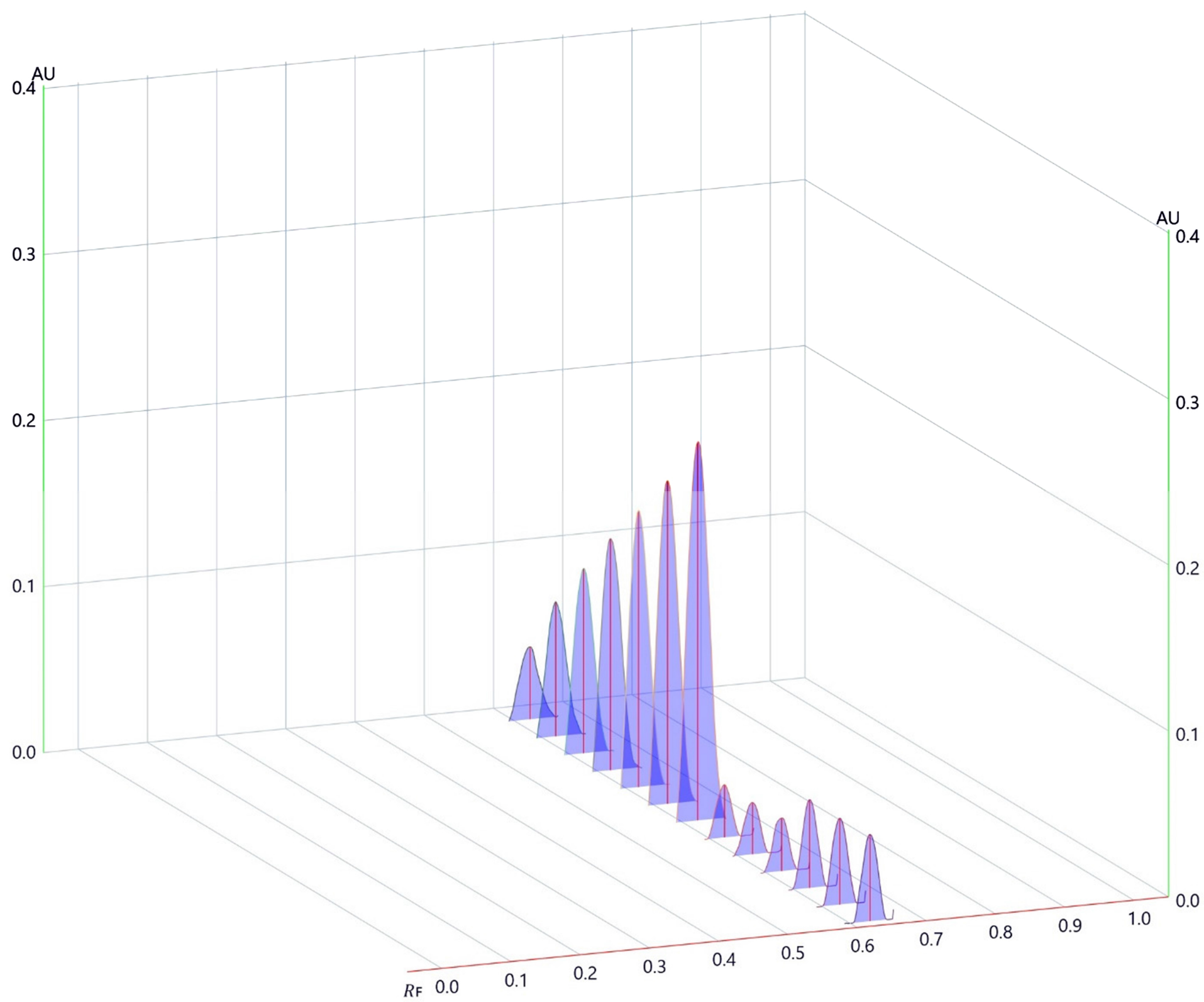
AU
0.4
0.3
0.2
0.1
0.0
RF
0.0
0.1
0.2
0.3
0.4
0.5
0.6
0.7
0.8
0.9
1.0

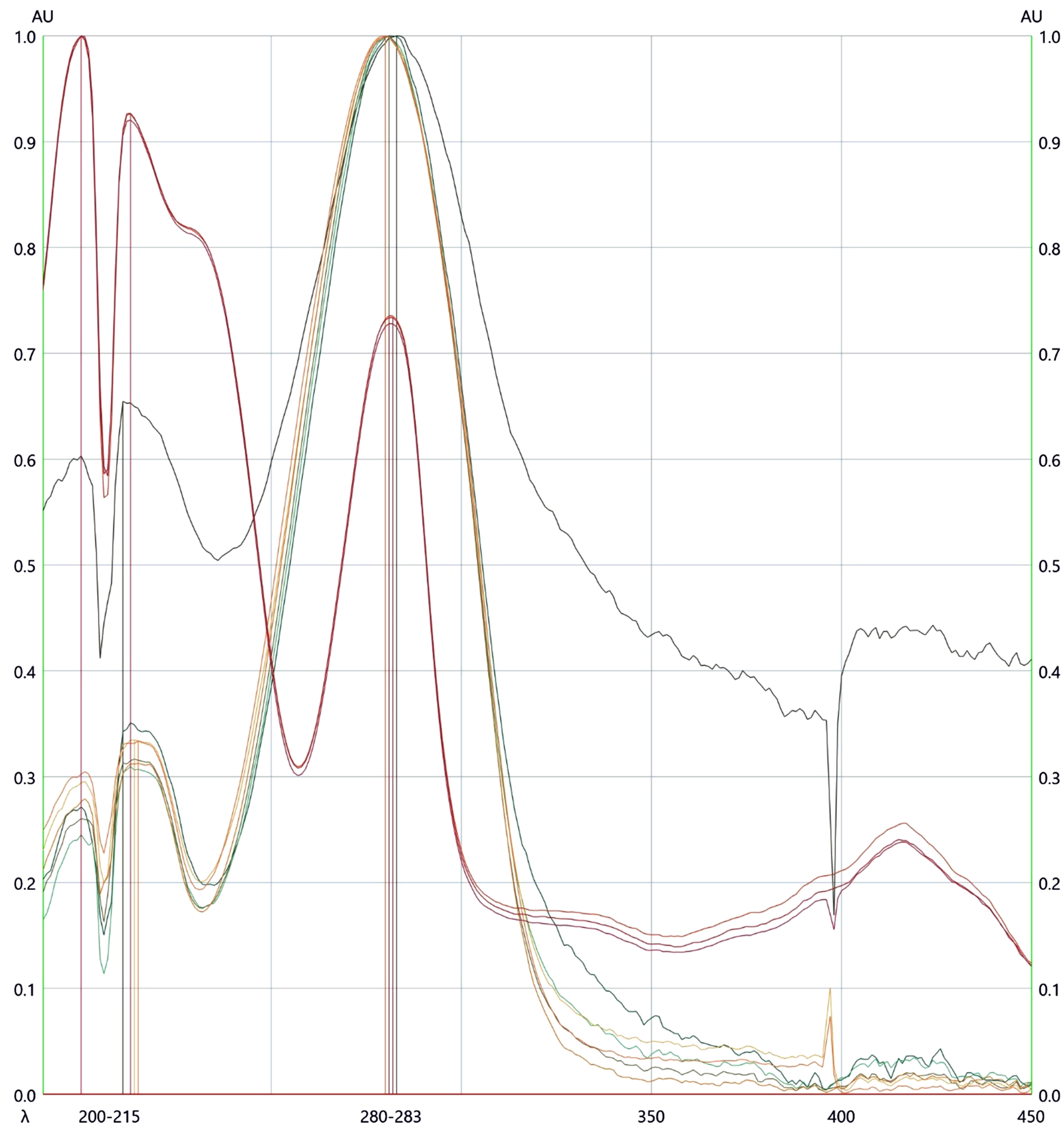
AU
1.0
0.9
0.8
0.7
0.6
0.5
0.4
0.3
0.2
0.1
0.0
AU
λ
200-215
280-283
350
400
450
